# Long noncoding RNA SALTe1, microvascular ageing, and cardiac dysfunction

**DOI:** 10.1093/eurheartj/ehag356

**Published:** 2026-05-19

**Authors:** Haobo Li, Xiao Xiao, Yirong Zhou, Yajing Zhang, Jiayi Kang, Zixun Yuan, Jiaqi He, Justin Guerra, Carolin Lerchenmüller, Kenneth B. Margulies, Aaron D. Aguirre, Jason D. Roh, Anthony Rosenzweig

**Affiliations:** 1 Department of Anesthesia, Critical Care, and Pain Medicine, Massachusetts General Hospital, 55 Fruit Street, Boston, MA 02114, USA; 2 Harvard Medical School, 25 Shattuck Street, Boston, MA 02115, USA; 3 Cardiovascular Research Center, Massachusetts General Hospital, Boston, MA 02114, USA; 4 Wellman Center for Photomedicine, Massachusetts General Hospital, Boston, MA 02114, USA; 5 Stanley and Judith Frankel Institute for Heart and Brain Health, University of Michigan Medical Center, North Campus Research Complex, 2800 Plymouth Road, NCRC Building 25, Ann Arbor, MI 48109-2800, USA; 6 Chair for Gender Medicine, University of Zurich, Zurich 8006, Switzerland; 7 Department of Cardiology, University Hospital Zurich, Zurich 8091, Switzerland; 8 German Center for Heart and Cardiovascular Research (DZHK), Heidelberg, Germany; 9 Department of Cardiology, University Hospital Heidelberg, Heidelberg 69120, Germany; 10 Cardiovascular Institute, Perelman School of Medicine, University of Pennsylvania, Philadelphia, PA 19104, USA

**Keywords:** Exercise, Ageing, Heart, Long noncoding RNA, Senescence, Endothelial cells

## Abstract

**Background and Aims:**

Ageing is accompanied by progressive microvascular dysfunction, a key determinant of organ performance and longevity. The molecular drivers of this process remain incompletely defined, and the mechanisms by which exercise counters vascular ageing are unclear. This study investigated whether exercise-regulated long noncoding RNAs (lncRNAs) contribute to microvascular ageing and age-related cardiac dysfunction.

**Methods:**

RNA sequencing was performed in naturally aged mice with and without voluntary exercise. Functional studies of candidate lncRNAs were conducted using AAV-mediated overexpression, antisense oligonucleotides, and CRISPR-based knockdown, both *in vivo* and in endothelial cells (ECs).

**Results:**

A novel set of lncRNAs altered in hearts from exercised aged mice was identified and termed Senescence Associated LncRNA Transcripts in Exercise (SALTes). Among these, SALTe1 is evolutionarily conserved and enriched in ECs. SALTe1 expression is elevated in hearts from aged mice and in patients with various types of heart failure but suppressed by exercise. In 20-month-old mice, SALTe1 inhibition, via antisense GapmeRs or EC-specific deletion, attenuated endothelial senescence, restored microvascular perfusion, and improved diastolic function. Mechanistic studies showed that SALTe1 acts, at least in part, through upregulation of PARP9.

**Conclusions:**

These findings establish SALTe1 as a pivotal regulator of endothelial senescence as well as microvascular and cardiac dysfunction in ageing. Targeted inhibition of SALTe1 recapitulates the vasculoprotective effects of exercise, highlighting a tractable antisense-based therapeutic strategy for combating age-related cardiac decline.

## Structured Graphical Abstract

**Key Question:**

Do exercise-regulated long noncoding RNAs contribute to microvascular ageing and age-related cardiac dysfunction?

**Key Finding:**

In mice, the endothelial long noncoding RNA SALTe1:

- was upregulated in microvascular endothelial cells in aged hearts and reduced by exercise.
- promoted microvascular ageing and age-related cardiac dysfunction, whereas its inhibition attenuated these phenotypes.
- regulated endothelial senescence in part through upregulation of PARP9.

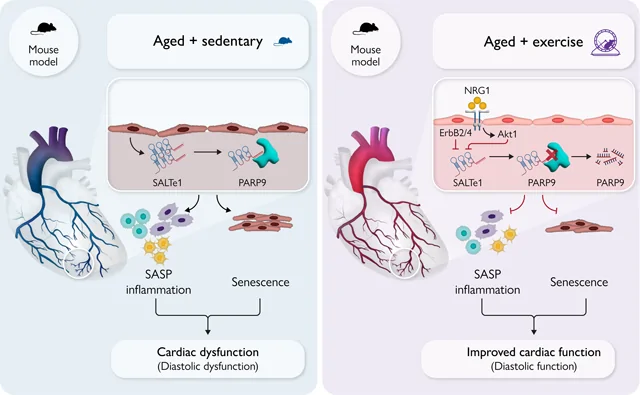

SALTe1 is upregulated in microvascular endothelial cells in aged hearts. SALTe1 promotes microvascular aging and age-related cardiac dysfunction by enhancing PARP9 mRNA stability, thereby inducing cardiac inflammation and endothelial senescence. Exercise attenuates microvascular aging and age-related cardiac dysfunction through NRG1-mediated downregulation of SALTe1

SASP, senescence-associated secretory phenotype

Li H, *et al. European Heart Journal.*

A long noncoding RNA SALTe1 regulates microvascular ageing and cardiac dysfunction.

## Introduction

Ageing-associated changes in microvascular density, structure, and function are key determinants of longevity and end-organ failure.^1–3^ In the heart, microvascular senescence and functional decline develop with age^3,4^ and are strongly associated with heart failure with preserved ejection fraction (HFpEF),^5^ the most common form of heart failure in older adults.^6^ Yet the mechanisms driving these age-related microvascular changes, their causal role in cardiac dysfunction, and their potential reversibility remain poorly defined.

Aerobic exercise has emerged as one of the most effective strategies for improving functional outcomes in older adults with HFpEF,^7^ leading some to suggest HFpEF represents an ‘exercise deficiency syndrome’.^8^ In preclinical models, exercise reverses hallmark features of age-related HFpEF, including impaired diastolic function, reduced cardiomyogenesis, and diminished capillary density.^9,10^ In both animals and humans, regular aerobic exercise prevents or reverses age-related microvascular changes in multiple tissues,^11–14^ including the heart.^13^ However, the molecular mechanisms by which exercise confers these vasculoprotective benefits are largely unknown.

Long noncoding RNAs (lncRNAs) have emerged as critical regulators of cardiovascular biology, with roles in development,^15^ hypertrophy,^16^ heart failure,^17^ atherosclerosis,^18^ and cardiovascular ageing.^18–20^ Our previous studies demonstrated that exercise is effective in improving cardiac function in the aged heart that is associated with broad transcriptional remodelling.^9,10^ However, whether lncRNAs also mediate the protective effects of exercise on microvascular ageing and age-related cardiac dysfunction has not been established.

Here, we identify a novel class of cardiac lncRNAs, we term SALTes (senescence associated lncRNA transcripts in exercise), that are dynamically regulated by exercise in old mice. Among them, SALTe1, a conserved lncRNA, was upregulated in aged hearts and downregulated by exercise. Functional studies revealed that SALTe1 promotes endothelial senescence, whereas systemic or endothelial-targeted inhibition of SALTe1 in aged mice attenuates vascular ageing and improves diastolic function. These findings establish a mechanistic link between exercise, endothelial senescence, and cardiac microvascular ageing, and implicate SALTe1 as a novel and potentially tractable therapeutic target for age-related microvascular and cardiac dysfunction.

## Methods

A detailed description of the methods and supporting data is available in the Supplementary Online Data. Related materials, data, and analytic methods are available from the corresponding author upon reasonable request.

### Animal experiments

All mice were maintained and studied using protocols in accordance with the NIH Guide for the Care and Use of Laboratory Animals and approved by MGH Animal Care and Use Committees (protocol numbers 2015N000029 and 2022N000209). Eight-to ten-week-old and 18-month-old male C57BL/6J mice were purchased from the Jackson Laboratory (Strain# 000664). Twenty-month-old C57BL/6J mice were provided by the National Institute on Ageing Rodent Colonies or purchased from the Jackson Laboratory. Rosa^Cas9GFP/Cas9GFP^ mice were purchased from the Jackson Laboratory (Strain# 026175).^16^ Eighteen-month-old male Rosa^Cas9GFP/Cas9GFP^ mice were used for studies.

### Running exercise protocol

Twenty-month-old mice were randomly assigned to running exercise as previously described.^10,16^ Briefly, mice were individually housed in plexiglass cages (36L × 20W × 15 H cm) that contained a stainless-steel running wheel (diameter 11.4 cm; Mini-Mitter, Starr Life Science, USA) equipped with a tachometer. Mice ran voluntarily for a total of eight weeks. Mouse activity was recorded daily. The sedentary control mice were kept in the same cage system lacking running wheels.

### Human samples

Procurement of human myocardial tissue was performed under protocols and ethical regulations approved by Institutional Review Boards at the University of Pennsylvania and the Gift-of-Life Donor Program (Pennsylvania, USA) as previously described.^2,3^ Failing hearts were obtained at the time of cardiac transplantation. Non-failing hearts (NF) were obtained at the time of organ donation from cadaveric donors. Written informed consent was obtained from all transplant recipients and from the authorized next-of-kin for deceased organ donors. *In vivo* function, based on left ventricle ejection fraction (LVEF), was assessed via echocardiography in all hearts. At the time of tissue procurement, all hearts were arrested *in situ* using ice-cold, blood-containing, high-potassium, cardioplegic solution. Explanted hearts were transported to the laboratory on wet ice in cold Krebs-Henseleit buffer (KHB) solution. Tissues were snap-frozen in liquid nitrogen within four hours and stored at −80°C until analysis.

### Statistical analysis

Data are presented as mean ± SEM unless otherwise indicated and analysed using GraphPad Prism 10 (GraphPad Software). Shapiro-Wilk test was used for data normality distribution analysis. Unpaired, two-tailed Student’s *t*-test was used for two-group comparisons. For multiple groups, one-way ANOVA with Dunnett’s *post hoc* test was used, and for grouped comparisons, two-way ANOVA with Tukey’s *post hoc* test was applied, as indicated. In Figure 2B, we used a pairwise Wilcoxon rank-sum test with Bonferroni correction. *P* < .05 was considered statistically significant.

## Results

### SALTe1 is downregulated by exercise in aged hearts

To identify lncRNAs that are dynamically regulated in aged hearts by exercise, RNA sequencing was performed on polyA-selected RNA from hearts of previously reported^10^ 20-month-old aged mice that underwent voluntary wheel running for eight weeks compared to age-matched mice under normal sedentary conditions (Figure 1A). Fourteen lncRNAs were significantly altered in exercised hearts (nine downregulated and five upregulated) (Figure 1A). Ten of these validated robustly by quantitative reverse transcription-polymerase chain reaction (QRT-PCR) in independent samples (Figure 1B and Supplementary data online, Table S1). We named these lncRNAs, Senescence-Associated LncRNA Transcripts in exercise (SALTes). Notably, in these SALTes, SALTe1 has been reported differentially expressed in hearts from 104-week-old mice compared with 14-week-old mice.^21^ Moreover, SALTe2 (1190002F15Rik) has been reported to promote the transcription of a senescence marker Cdkn1b in erythroid cells.^22^ In addition, SALTe4 (Gm45670) has been reported to regulate smooth muscle cell phenotypic switch,^23^ indicating the robustness of our strategy for identifying lncRNAs involved in microvascular ageing and cardiac ageing. Among the validated SALTes, we focused on the four SALTes (SALTe1, 2, 5, and 9) that were downregulated by exercise, suggesting that antisense inhibition of these SALTes could mimic the benefits of exercise. Of these, SALTe1 was increased in aged hearts, whereas SALTe5 and 9 were decreased. SALTe2 level remained stable in aged hearts relative to young controls. Among these four SALTes, three (SALTe1, 2, and 9) were robustly expressed in cardiac ECs compared to other cell types in the heart, including cardiomyocytes (CMs) and fibroblasts (FBs) (Figure 1C). Previous studies suggest that regular aerobic exercise is effective in preventing or reversing age-related microvascular changes across various tissues,^11–14^ prompting us to focus on three SALTes that are robustly expressed in ECs of aged hearts.

In ECs isolated from 20-month-old mouse hearts, transfection with locked nucleic acid (LNA) antisense oligonucleotide (ASO) GapmeRs specific to SALTe1, 2, or 9 led to ≈55% reductions in SALTe1, 2, and 9 RNA levels (see Supplementary data online, Figure S1A), similar to the reduction seen in exercised hearts (Figure 1B). Knockdown of SALTe1 and SALTe9 reduced expression of senescence genes including Cdkn2a, Cdkn1a, and Serpine1, while knockdown of SALTe2 reduced gene expression of Cdkn1a and Serpine1 but not Cdkn2a (Figure 1D). Knockdown of SALTe1 and 2 decreased β-galactosidase positive cells, and increased telomere length as well as increasing the number of EdU-positive EC as a marker of cell proliferation (Figure 1E and F and Supplementary data online, Figure S1C). Similar effects were seen with SALTe9 knockdown except that it did not change telomere length (Figure 1D–F and Supplementary data online, Figure S1C). These results suggest a reduction in cellular senescence after SALTe1, 2, and 9 knockdown. In cardiac ECs isolated from 10-week-old mice, overexpression of SALTe1, 2, and 9 with a CRISPR/dCAS9 activation system^16^ increased SALTe1, 2, and 9 expression by ~2.3-fold, ~1.7-fold, and ~2.2-fold, respectively (see Supplementary data online, Figure S1B). Overexpression of SALTe1 and 2 increased expression of senescence genes Cdkn2a, Serpine1, and Cdkn1a, while overexpression of SALTe9 increased expression of Cdkn2a and Cdkn1a but not Serpine1 (Figure 1G). Overexpression of SALTe1 and 2, but not 9, increased β-galactosidase positive cells, and reduced telomere length (Figure 1H and I). Only SALTe1 overexpression reduced cell proliferation (number of EdU positive cells) (see Supplementary data online, Figure S1D). Together, these data suggest that SALTe1 and 2 are necessary and sufficient for EC senescence. Of these lncRNAs, SALTe2 was not changed in aged hearts compared with young hearts (Figure 1B). Moreover, in a published study,^24^ SALTe1 expression showed a nonsignificant trend to increase in ECs isolated from 20-month-old hearts compared with 3-month-old mice. A similar trend in ECs of aged hearts was observed in a recent single-cell sequencing study.^25^ We, thus, selected SALTe1 for further investigation because it had the most consistent effects on cardiac EC senescence in our study.

### SALTe1 is conserved between mouse and human

The full-length SALTe1 sequence (ENSMUST00000173605) was confirmed by rapid amplification of cDNA ends, which was the only transcript isoform detected in the heart among the three annotated isoforms. SALTe1 is conserved between mouse and human in both sequence and genomic location. The mouse ortholog of SALTe1 exhibits ~27% nucleotide identity with its human counterpart (ENSG00000287207) (Figure 2A). In both species, *Parp9* and *Kpna1* are the two closest protein-coding genes (Figure 2A). SALTe1 is also conserved at the sequence level across multiple species (see Supplementary data online, Figure S2A). In hearts explanted from patients with dilated cardiomyopathy^26^ (DCM, *n* = 12), hypertrophic cardiomyopathy^26^ (HCM, *n* = 10), or ischemic cardiomyopathy^27^ (ICM, *n* = 11), SALTE1 levels were increased in all these three types of heart failure compared with nonfailing controls^26^ (NF, *n* = 10) (Figure 2B). No significant association between age and SALTE1 levels was observed due to the limited number of patients with available age information (data not shown). To assess whether SALTe1 has similar functional effects in human ECs, we examined the role of human SALTe1 in human cardiac microvascular endothelial cells (HMVECs) that had undergone replicative senescence induced by 18 serial passages.^28^ Compared to passage four, SALTE1 levels were increased in passage 18 HMVECs (Figure 2C). In senescent HMVECs, inhibition of human SALTe1 by LNA-GapmeR reduced expression of the senescent gene markers (Cdkn2a, Cdkn1a, and TP53) and the number of SA-β-gal positive HMVECs (Figure 2D–F). In contrast, in non-senescent HMVECs passaged only four times, lentiviral overexpression of human SALTe1 increased expression of the senescence markers, Cdkn2a and Cdkn1a, and the number of SA-β-gal positive HMVECs (see Supplementary data online, Figure S2B and C). Notably, in *mouse* cardiac ECs isolated from 10-week-old mice, lentiviral overexpression of *human* SALTE1 also increased expression of the senescence markers, Cdkn2a, Cdkn1a, and TP53, and β-galactosidase positive cells (Figure 2G and H). Taken together, these data demonstrate that both mouse and human SALTe1 regulate EC senescence and that its structural conservation is sufficient to enable conservation of this function across species.

The Coding Potential Assessment Tool^29^ failed to identify any major open reading frames (ORF) with translational potential longer than 100 amino acids in mouse SALTe1. However, one small ORF (sORF) was identified in SALTe1. To test whether this sORF has peptide-coding potential, full-length mouse SALTe1 or the sORF was cloned upstream of the 3xFLAG coding sequence in p3xFLAG-CMV and transfected into HEK293T cells. No FLAG-tagged peptides were detected, suggesting that this sORF is not translated at a significant level (Figure 2I). To further examine this important question, RNA was immunoprecipitated with an antibody against ribosome protein L4 (RPL4), a ribosome subunit required for translation elongation.^30^ Immunoprecipitation worked well (Figure 2J, upper) and the immunoprecipitated complex was highly enriched for the positive control GAPDH mRNA but there was no enrichment for SALTe1 or the CHAER lncRNA, used as a negative control (Figure 2J). These data strongly suggest that SALTe1 does not encode translated protein.

SALTe1 is expressed by several tissues, including the heart, in which its expression is higher in ECs (see Supplementary data online, Figure S2D). In both murine and human MVECs, SALTe1 predominantly localized to the nucleus (see Supplementary data online, Figure S2E and F). To examine the upstream regulators of SALTe1, cardiac ECs were isolated from 20-month-old mice and exposed to stimuli that positively (NRG1, IGF-1, or ET-1) or negatively (H_2_O_2_, LPS, TNFα, or doxorubicin^31^) modulate EC function, senescence, and cell survival in ECs. Among them, NRG1 reduced expression of SALTe1 and senescence genes (Figure 2K and Supplementary data online, Figure S2G), while IGF-1 showed a nonsignificant trend towards reducing SALTe1 expression (Figure 2K). NRG1-induced SALTe1 downregulation was attenuated by knockdown of either the individual receptor components (ErbB2 or ErbB4) or the downstream signalling effector of this pathway while knockdown of ErbB3 had no effect (Figure 2L), suggesting that NRG1 inhibits SALTe1 through Akt1, ErbB2, and ErbB4. In contrast, treatment with lipopolysaccharide (LPS) increased expression of SALTe1 and senescence genes (Figure 2M and Supplementary data online, Figure S2H). LPS-induced SALTe1 was reversed by toll-like receptor 4 gene knockdown (Figure 2M), indicating that LPS upregulates SALTe1 through toll-like receptor 4. Moreover, SALTe1 expression was increased by doxorubicin, an anthracycline chemotherapy agent that induces cellular senescence^32^ (Figure 2K). Among the myokines involved in exercise and examined (NRG1, VEGFa, IGF-1, FGF2, and PDGFα), cardiac NRG1 mRNA exhibited the greatest fold change in 20-month-old mice after eight weeks of voluntary wheel exercise (see Supplementary data online, Figure S2I). Moreover, cardiac NRG1 mRNA expression was increased after exercise in both 10-week-old and 20-month-old mice (Figure 2N). Together, these results suggest that exercise inhibits SALTe1 through NRG1.

To identify the optimal GapmeR for *in vivo* experiments, we tested two additional GapmeRs (GapmeR#2 and #3), in addition to the previously validated GapmeR#1. In primary cardiac ECs from 20-month-old mice, transfection with three LNA GapmeRs (GapmeR #1, #2, and #3) specific to different regions of SALTe1 each led to a ≈55% reduction in SALTe1 transcriptional levels (see Supplementary data online, Figure S3A). SALTe1 knockdown reduced expression of senescence genes (Cdkn2a, Cdkn1a, TP53, and Serpine1) as well as increasing EC migration and tube formation (see Supplementary data online, Figure S3B and C). These results indicate that inhibition of SALTe1 attenuates cellular senescence and improves angiogenic EC function *in vitro*. Based on its greater effect size on these assays, GapmeR #1 was chosen for inhibiting SALTe1 *in vivo* in mice studies.

### SALTe1 overexpression induces endothelial ageing and cardiac dysfunction

To examine the effects of SALTe1 expression in cardiac ECs *in vivo*, EC-enhanced AAV (AAV^endo^) encoding EC-specific CD144 promoter-driven SALTe1 was generated, which utilizes a modified capsid by inserting AEGDVAR peptide to AAV2, which previously shown to have high efficacy and specificity in transducing cardiac ECs *in vivo* through directed evolution capsid screening.^33^ In hearts from mice four weeks after injection of AAV^endo^ encoding an EC promoter–driven GFP, flow cytometry showed that GFP-positive cells were observed primarily in ECs (CD31-positive and CD45-negative), but not in immune cells (CD45-positive) or fibroblasts (MEFSK4-positive) (see Supplementary data online, Figure S4A and B), indicating the specificity of AAV^endo^ in transducing cardiac ECs. AAV^endo^-CD144-SALTe1 was injected via the tail vein of 8-week-old wild-type mice (Figure 3A). Twelve weeks after injection, AAV^endo^-CD144-SALTe1 led to a ~5-fold increase in cardiac SALTe1 expression compared to AAV^endo^-CD144-Control (Figure 3B), without impacting SALTe1 expression and senescence genes in the skeletal muscle (see Supplementary data online, Figure S4C and D). This induction of EC SALTe1 increased E/e’, decreased cardiac contractile reserves and global longitudinal strain (GLS) (Figure 3C–E), but did not affect resting fractional shortening (FS) (Figure 3F and Supplementary data online, Table S2). Cardiac EC SALTe1 overexpression did not affect heart weight (HW) to tibial length (TL) but increased lung weight (LW) to TL ratio (Figure 3G and H). These suggest an induction of diastolic dysfunction and subclinical systolic dysfunction (GLS) by SALTe1 overexpression. Moreover, EC SALTe1 overexpression increased gene expression of senescence (Cdkn2a, Cdkn1a, TP53, and Serpine1) and pathological cardiac hypertrophy (Nppa and Nppb) in the heart (Figure 3I). These were associated with decreased cardiac capillary density (decreased CD31-positive cells) and increased cardiac EC senescence (increased CD31-Cdkn2a^INK4A^ or −γH2AX double-positive cells) (Figure 3J–M). These results demonstrate that cardiac EC SALTe1 overexpression is sufficient to induce EC senescence and cardiac dysfunction.

### SALTe1 inhibition attenuates microvascular ageing in aged hearts

To examine the effects of SALTe1 inhibition on vascular ageing in the heart *in vivo*, aged (20-month-old) mice were injected with either scrambled control GapmeR or SALTe1-specific GapmeR #1 subcutaneously for four weeks (Figure 4A). Ten-week-old mice were used as young controls. Compared to young mice, aged mice had increased cardiac SALTe1 expression (Figure 4B) and decreased myocardial perfusion demonstrated by an increased time-to-peak (TTP) measured by myocardial contrast echocardiography (Figure 4C). These changes were associated with reduced cardiac capillary density (decreased CD31-positive cells), increased cardiac EC senescence (increased CD31-Cdkn2a^INK4A^ or −γH2AX double-positive cells) (Figure 4D–G), increased senescence-associated secretory phenotype (SASP) gene expression, and a reduction in pro-angiogenic gene expression (Figure 4H and I). Inhibition of SALTe1 for 4 weeks led to ~60% decrease in cardiac SALTe1 expression (Figure 4B). Remarkably, inhibition of SALTe1 improved myocardial perfusion, increased cardiac capillary density, reduced cardiac EC senescence, and reduced SASP gene expression while increasing pro-angiogenic gene expression (Figure 4C–I). Most of these parameters were reversed to levels comparable to those seen in young animals. Thus, inhibition of SALTe1 mitigates many hallmarks of microvascular ageing in the heart.

### SALTe1 inhibition improves cardiac function in aged hearts

We then examined whether SALTe1 inhibition and mitigating vascular ageing had an impact on myocardial structure and function (Figure 5A). Although FS was not significantly different in aged mice (Figure 5B), aged mice exhibited diastolic dysfunction, including increased E/e’ and decreased cardiac contractile reserves (Figure 5C and D and Supplementary data online, Table S3), as well as evidence of subclinical systolic dysfunction with reduced GLS (Figure 5E). Compared to young mice, aged mice had increased HW/TL and LW/TL (Figure 5F and G), suggestive of pulmonary congestion. Inhibition of SALTe1 increased cardiac contractile reserves and GLS while decreasing E/e’ (Figure 5C–E and Supplementary data online, Table S3), consistent with mitigation of diastolic and subclinical systolic dysfunction. Inhibition of SALTe1 also decreased LW/TL without affecting HW/TL (Figure 5F and G), suggesting mitigation of pulmonary congestion and heart failure without a change in cardiac hypertrophy.

Since GapmeR treatment inhibits SALTe1 systemically, it is possible that some of the cardiac benefits observed derive from SALTe1 inhibition in the peripheral vasculature or even in other lineages where it might be expressed at very low levels. To examine the role of SALTe1 specifically in cardiac ECs, we generated a cardiac EC-targeted SALTe1 loss-of-function model using CAS9 knock-in mice^16^ and the cardiac EC-enhanced AAV^endo^, which, as shown above (see Supplementary data online, Figure S4), specifically targets cardiac EC. AAV^endo^ encodes U6-driven guide RNAs (gRNAs) targeting exon 1 (gRNA1) of SALTe1 followed by Cre under control of the EC-specific promoter, Tie2 (Figure 5H). gRNA1 or scrambled control was packaged in AAV^endo^ and injected into the tail veins of 18-month-old CAS9 knock-in mice. Twelve weeks after injection, AAV^endo^-SALTe1-gRNA1 led to a ~60% reduction in cardiac SALTe1 expression compared to AAV^endo^-control-gRNAs (Figure 5I). AAV^endo^-SALTe1-gRNA1 injection did not affect SALTe1 expression in the skeletal muscle and liver (see Supplementary data online, Figure S4E and G), in contrast to systemic SALTe1 inhibition by GapmeR, which led to ~60% reduction of SALTe1 in the skeletal muscle after 4 weeks of injection (see Supplementary data online, Figure S4F), confirming that AAV^endo^-SALTe1-gRNA1 specifically inhibits SALTe1 in cardiac EC. AAV^endo^-mediated cardiac EC SALTe1 deletion did not change FS (Figure 5J) but resulted in increased GLS and cardiac contractile reserves while decreasing E/e’ (Figure 5K–M and Supplementary data online, Table S4). Importantly, deletion of cardiac EC SALTe1 resulted in reduced LW/TL without affecting HW/TL (Figure 5N and O), demonstrating that inhibition of SALTe1 specifically in cardiac ECs, improves cardiac diastolic and subclinical systolic dysfunction. Taken together, these data demonstrate that endothelial SALTe1 expression is necessary for EC senescence, multiple hallmarks of microvascular remodelling, and diastolic dysfunction in the ageing heart and represents a tractable therapeutic target in this context.

Senolytics have demonstrated efficacy in inhibiting vascular ageing and improving cardiac function in aged animals.^34–36^ To explore whether SALTe1 plays a role in mediating senolytics’ anti-ageing effects in the heart, 20-month-old mice were treated with the senolytic, fisetin, for three weeks (see Supplementary data online, Figure S5A). Fisetin treatment improved diastolic function, including reduced E/e’ and increased cardiac contractile reserves (see Supplementary data online, Figure S5B and C), as well as elevated GLS (see Supplementary data online, Figure S5D). Compared to aged mice treated with vehicle control, fisetin treatment reduced LW/TL while modestly affecting HW/TL in aged mice (see Supplementary data online, Figure S5E and F). Moreover, in cardiac ECs isolated from aged mice treated with fisetin, gene expression of senescence markers Cdkn2a, Cdkn1a, TP53, and Serpine1, as well as SALTe1 were reduced compared to ECs isolated from hearts of aged mice treated with vehicle control (see Supplementary data online, Figure S5G and H). These results provide additional support for the role of SALTe1 in EC microvascular ageing and remodelling.

### SALTe1 regulates endothelial cell function through PARP9

lncRNAs often regulate expression of nearby genes, so we focused first on potential target genes located close to the genomic region encoding SALTe1. *Parp9* (poly (ADP-ribose) polymerase 9) and *Kpna1* (karyopherin Subunit Alpha 1) are the two nearest genomic neighbours to SALTe1 in both mice and humans (Figure 2A). In cardiac EC isolated from aged (20-month-old) mice, SALTe1 gene knockdown reduced PARP9 but not KPNA1 gene expression (Figure 6A). Moreover, in cardiac ECs isolated from young (8-week-old) mice, lentiviral overexpression of SALTe1 increased (Figure 6B, left panel), while inhibition of SALTe1 in cardiac EC from aged mice decreased PARP9 protein expression (Figure 6B, right panel). suggesting that SALTe1 regulates PARP9 at the mRNA and protein levels.

Since portions of PARP9 and SALTe1 overlap and are transcribed from opposite strands (Figure 2A), we examined whether SALTe1 binds to PARP9 mRNA and regulates PARP9 mRNA stability. Mouse cardiac ECs were treated with control or lentiviral SALTe1 overexpression, followed by cross-linking, total RNA extraction, and pulldown using antisense probes complementary to SALTe1, or sense probes (as negative controls), or a no-probe control. RT-QPCR analysis of the pulled-down fractions revealed a ~2-fold increase in the enrichment of PARP9 mRNA in the antisense probe group compared to the sense and no-probe controls (Figure 6C). This PARP9 mRNA enrichment was further increased in ECs overexpressing SALTe1 (Figure 6C). These results suggest that SALTe1 directly binds to PARP9. To examine whether this binding affects PARP9 mRNA stability, total RNA was extracted from mouse cardiac ECs and RT-PCR-amplified with primers specific for the non-overlapping and overlapping portions of PARP9 and SALTe1, and levels were not changed by lentiviral SALTe1 overexpression (Figure 6D, left 4 bars). As expected, after treatment with RNase A to remove single-stranded RNA, RT-QPCR only detected overlapping regions of SALTe1 and PARP9, and this signal was further enhanced by lentiviral SALTe1 overexpression (Figure 6D, right 4 bars). These data suggest that SALTe1 binds PARP9 forming an RNA duplex and protecting it from RNAse A. In ECs treated with actinomycin D to suppress transcription, lentiviral SALTe1 overexpression increased the half-life of PARP9 mRNA (Figure 6E), suggesting SALTe1 promotes PARP9 mRNA stability by forming an RNA duplex.

PARP9 has previously been described to regulate NAD metabolism^37^ and pro-inflammatory gene expression,^38^ two processes relevant to cardiovascular ageing.^39,40^ To examine whether PARP9 contributes to the vascular effects of SALTe1, we manipulated SALTe1 and PARP9 in ECs isolated from aged mice. Lentiviral overexpression of PARP9 in aged cardiac EC attenuated SALTe1 inhibition-induced downregulation of senescence gene expression (Cdkn2a, Cdkn1a, and TP53) and improvement of tube formation (Figure 6F and G). Consistently, in low-passage HMVECs (P4), lentiviral overexpression of human SALTE1 increased PARP9 protein expression (Figure 6H). Conversely, in HMVECs where senescence was induced by repeated passaging (P18), SALTE1 gene knockdown reduced PARP9 protein expression (Figure 6H). Moreover, SALTE1 gene knockdown in these senescent HMVECs (P18) reduced senescence gene expression and improved tube formation (Figure 6I and J). These effects were reversed by lentiviral overexpression of PARP9 (Figure 6I and J). These results suggest that SALTe1 induces EC senescence and impairs angiogenesis, in part by increasing expression of PARP9, likely through enhanced PARP9 mRNA stability.

### PARP9 inhibition improves cardiac function in aged hearts

In cardiac ECs isolated from 20-month-old mice, inhibition of PARP9 by siRNA reduced expression of senescence gene markers (Cdkn2a, Cdkn1a, and Serpine1), increased telomere length, and enhanced tube formation (see Supplementary data online, Figure S6A–C). To investigate the functional role of PARP9 in vascular ageing in the heart *in vivo*, we delivered cardiac EC enhanced AAV^endo33^ carrying U6 promoter-driven shRNA targeting PARP9 to 20-month-old mice (Figure 7A). Nine weeks after AAV^endo^ injection, compared to mice injected with AAV^endo^-scrambled control, AAV^endo^-PARP9-shRNA led to ~55% downregulation of PARP9 in the heart (Figure 7B) but not the skeletal muscle (see Supplementary data online, Figure S6D). This reduction of PARP9 did not affect HW/TL or LW/TL (Figure 7C and D). Inhibition of PARP9 did not change FS but reduced E/e’, increased GLS, and improved cardiac contractile reserves (Figure 7E–H and Supplementary data online, Table S5), suggesting a mitigation of diastolic and subclinical systolic dysfunction. Inhibition of PARP9 also reduced cardiac expression of senescence gene markers, Cdkn2a and Cdkn1a, without affecting TP53 or Serpine1 (Figure 7I). RNA-sequencing from ECs with or without PARP9 gene knockdown revealed that PARP9 knockdown altered genes enriched in pathways associated with senescence (e.g. FoxO and TP53 signalling pathways) and angiogenesis (e.g. PI3K-Akt and HIF-1 signalling pathways) as well as NAD metabolism (e.g. Citrate cycle [TCA cycle] pathway) (Figure 7J), supporting an important role for PARP9 in regulating EC senescence and function in aged hearts. Together, these data support the model that SALTe1 induces EC senescence at least in part by increasing PARP9 expression.

## Discussion

Exercise is one of the few interventions known to improve functional capacity and quality of life in older adults. Aerobic exercise has emerged as an effective strategy for preventing age-related vascular changes and improving cardiac performance in older adults,^7^ and aerobic exercise has consistently been shown to mitigate vascular ageing and enhance cardiac performance. In preclinical models, multiple exercise regimens, including swim training, voluntary wheel running, and treadmill exercise, reduce coronary endothelial cell (EC) senescence, promote cardiomyogenesis, and increase capillary density, with corresponding improvements in cardiac function.^9,10,13^ These findings underscore the effectiveness of exercise in counteracting age-related vascular and cardiac changes. Yet the molecular mechanisms underlying these benefits have remained largely undefined, particularly the role of lncRNAs in this context.

Here we identify a novel set of exercise-regulated cardiac lncRNAs, termed **S**enescence-**A**ssociated **L**ncRNA **T**ranscripts in **E**xercise (SALTes). Among these, SALTe1 emerged as a key regulator of endothelial senescence, that was structurally and functionally conserved across species. SALTe1 was upregulated in hearts from aged mice and patients with heart failure, and downregulated by exercise. In young mice, SALTe1 cardiac EC overexpression was sufficient to induce endothelial senescence and cardiac dysfunction. Conversely, systemic or EC-specific inhibition of SALTe1 in aged mice mitigated many hallmarks of microvascular ageing, including improvement in myocardial perfusion and capillary density, and reduced EC senescence. Importantly, inhibition of endothelial SALTe1 also restored diastolic function in aged mice. Moreover, treatment with the senolytic, fisetin, reduced EC senescence and improved cardiac function in aged mice as well as reducing EC SALTe1 expression. These findings position SALTe1 as a central mediator of vascular and cardiac ageing.

Although EC senescence has been proposed as a contributor to cardiac dysfunction, including age-related diastolic dysfunction,^35,41–43^ a causal role for EC senescence in age-related cardiac dysfunction and whether it can be targeted therapeutically have not been established. Our results provide direct evidence that targeting endothelial senescence, specifically through inhibition of SALTe1, rescues age-related microvascular decline and cardiac dysfunction, likely through the improved angiogenesis and microvascular perfusion, reducing secretion of SASP proteins, key drivers of diastolic dysfunction in the aged heart.^35,44^ Both systemic antisense treatment and cardiac EC-specific knockdown using an AAV-CAS9 system^16^ produced comparable improvements in diastolic function, strongly supporting the conclusion that endothelial senescence is a driving mechanism of cardiac ageing and a modifiable therapeutic target.

We also identify PARP9 as a downstream effector of SALTe1 in promoting EC senescence and regulating age-related cardiac dysfunction. While PARP9 has been implicated in inflammatory and DNA damage responses,^45–47^ its role in the heart was unknown. We show that PARP9 is upregulated in cardiac ECs with ageing, and that EC-specific knockdown improves diastolic function in aged mice. Transcriptomic analyses further implicate PARP9 in pathways of senescence and angiogenesis, suggesting roles beyond proinflammatory regulation. Although our study focused on cardiac ECs, PARP9 is also expressed in other lineages, including macrophages,^46^ which are known contributors to cardiac ageing. Defining the contribution of PARP9 in these non-endothelial compartments will be an important direction for future work.

In summary, we identify the SALTe1/PARP9 pathway as a novel and tractable regulator of microvascular ageing and age-related cardiac dysfunction. These findings establish endothelial senescence as a causal driver of functional decline in the ageing heart and suggest that therapeutic targeting of this pathway may offer a new strategy to combat not only cardiac ageing, but potentially a broader spectrum of age-related vascular and cardiac diseases.

Several limitations of this study warrant consideration. First, although our data suggest that PARP9 is a primary downstream effector of SALTe1 in ECs, lncRNAs often function through complex *cis*- and *trans*-regulatory networks.^48^ It is possible that SALTe1 regulates microvascular ageing through additional, yet-to-be-identified pathways. Second, while we focused on stimuli that regulate EC function and exercise-induced myokines as upstream regulators, SALTe1 expression may also be modulated by transcriptional regulation via promoter binding^49^ or internal RNA modifications.^50^ Third, while the observation that SALTe1 is increased in patients with multiple types of heart failure is exciting and suggests its potential as a therapeutic target, these data were obtained from whole-heart tissues and therefore cannot exclude contributions from non-endothelial populations. Although SALTe1 is expressed at lower levels in other (non-EC) cardiac cell populations, this does not exclude biological roles in these cells. Similarly, while we used an EC-enhanced AAV capsid to improve targeting specificity, off-target contributory effects in other cell types cannot be completely excluded. Additionally, the role of SALTe1 as a general driver of senescence across other tissues remains unclear, as our focus was restricted to the EC. Lastly, we prioritized naturally ageing animal models to ensure clinical relevance to human cardiac ageing. While accelerated ageing models like Tert knockout or Lmna^G609G^ mice are valuable, they often manifest distinct pathologies, such as cardiac atrophy,^51^ that differ from the hypertrophy characteristic of natural human ageing.

## Supplementary Material

Supplemnentary material

Supplementary data

Supplementary data are available at *European Heart Journal* online.

## Figures and Tables

**Figure 1:**
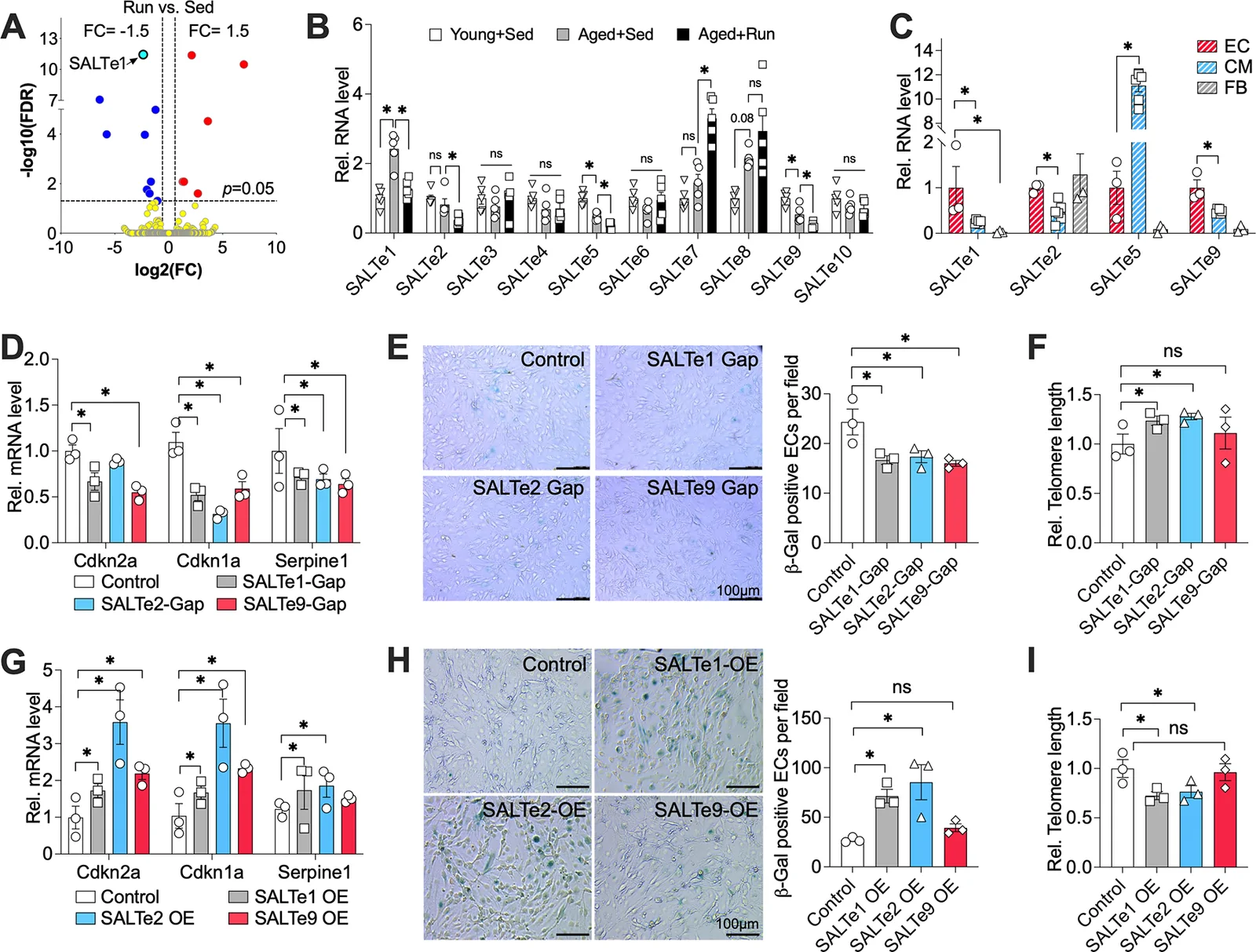
SALTe1 is downregulated by exercise in aged hearts. **A**, Volcano plot showing IncRNA RNA sequencing results in hearts from aged running (Run) mice compared with sedentary (Sed) mice; *n* = 4 mice/group. **B**, Quantitative reverse transcription-polymerase chain reaction (QRT-PCR) measurement of SALTes in young mice with sedentary, aged mice with sedentary (Sed) or running (Run); *n* = 5 mice/group. **C**, mRNA expression of SALTe1, 2, 5, and 9 in endothelial cells (EC), cardiomyocytes (CM), or fibroblasts (FB) isolated from hearts; *n* = 3–6 mice/group. **D**, mRNA expression of Cdkn2a, Cdkn1a, and Serpine1 from ECs treated with scrambled control (control) or SALTe1, 2, and 9 specific GapmeRs; *n* = 3 independent biological replicates/group. **E**, Representative and quantification of β-galactosidase (β-gal) staining; *n* = 3 independent biological replicates/group. **F**, Telomere length measured by QRT-PCR; *n* = 3 independent biological replicates/group. **G**, mRNA expression of Cdkn2a, Cdkn1a, and Serpine1 from ECs treated with control vector (control) or SALTe1, 2, and 9 lentiviruses (OE); *n* = 3 independent biological replicates/group. **H**, Representative and quantification of β-galactosidase (β-gal) staining; *n* = 3 independent biological replicates/group. **I**, Telomere length measured by QRT-PCR; *n* = 3 independent biological replicates/group. Data shown as mean ± SEM. *P* < .05 by One-way ANOVA with Dunnett’s *post hoc* test; ns, not significant

**Figure 2:**
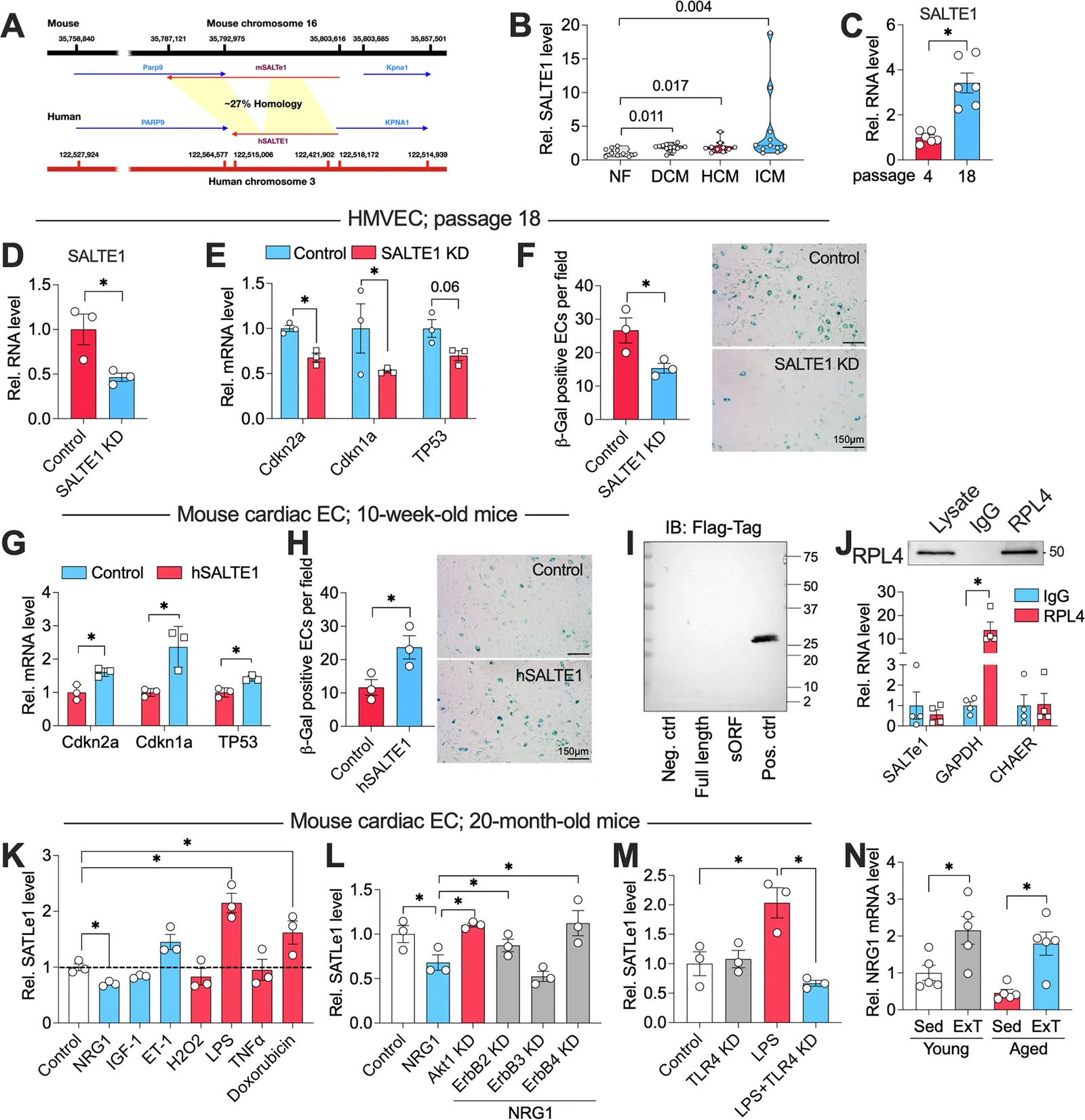
SALTe1 is conserved between mouse and human. **A**, Schematic diagram of SALTe1 in mouse and human genomes. **B**, SALTE1 RNA levels in cardiac tissues from patients with dilated cardiomyopathy (DCM, *n* = 12), hypertrophic cardiomyopathy (HCM, *n* = 10), ischaemic cardiomyopathy (ICM, *n* = 11), or nonfailing controls (NF, *n* = 10). **C**, SALTE1 RNA level in human cardiac microvascular endothelial cells (HMVECs) passage number 4 and 18. **D**, SALTE1 RNA level in HMVECs passage number 18 treated with scrambled control (control) or SALTe1 specific GapmeR (KD); *n* = 3 independent biological replicates/group. **E**, Gene expression of Cdkn2a, Cdkn1a, and TP53 in HMVECs; *n* = 3 independent biological replicates/group. **F**, Representative and quantification of β-galactosidase (β-gal) staining; *n* = 3 independent biological replicates/group. **G**, mRNA expression of Cdkn2a, Cdkn1a, and TP53 in ten-week-old mouse cardiac micro-vascular endothelial cells (ECs) treated with lentiviruses expressing control vector (control) or human SALTe1 (hSALTE1); *n* = 3 independent biological replicates/group. **H**, Representative and quantification of β-galactosidase (β-gal) staining; *n* = 3 independent biological replicates/group. **I**, Representative image of western blot from HEK293T transfected with negative control (neg.ctrl), full length of SALTe1 (Full length), small ORF identified within SALTe1 (sORF), or positive control (pos.ctrl). **J**, Gene expression of SALTe1, GAPDH, and CHAER in lysate pulldown with RPL4 antibody; *n* = 4 independent biological replicates/group. **K**, SALTe1 RNA expression in ECs isolated from 20-month-old mice and treated with DMSO (control), human NRG1 recombinant, human IGF-1, endothelin-1 (ET-1), H_2_O_2_, lipopolysaccharide (LPS), TNFα, and doxorubicin; *n* = 3 independent biological replicates/group. **L**, SALTe1 RNA expression in ECs isolated from 20-month-old mice and treated with DMSO (control), NRG1 with or without Akt1 siRNA (Akt1 KD), ErbB2 siRNA (ErbB2 KD), ErbB3 siRNA (ErbB3 KD), or ErbB4 siRNA (ErbB4 KD); *n* = 3 independent biological replicates/group. **M**, SALTe1 RNA expression in ECs treated with DMSO (control), Toll-like receptor 4 siRNA (TLR4 KD), LPS, or their combination.; *n* = 3 independent biological replicates/group. **N**, NRG1 mRNA levels in hearts from young and aged mice with sedentary (Sed) or eight-weeks of voluntary wheel running (E×T); *n* = 5 mice/group. Data shown as mean ± SEM. *P < .05 by pairwise Wilcoxon rank-sum test with Bonferroni correction in B; by unpaired two-tailed Student’s *t*-test in C-J, by One-way ANOVA with Dunnett’s *post hoc* test in K-M, and by Two-way ANOVA with Tukey’s *post hoc* test in N

**Figure 3:**
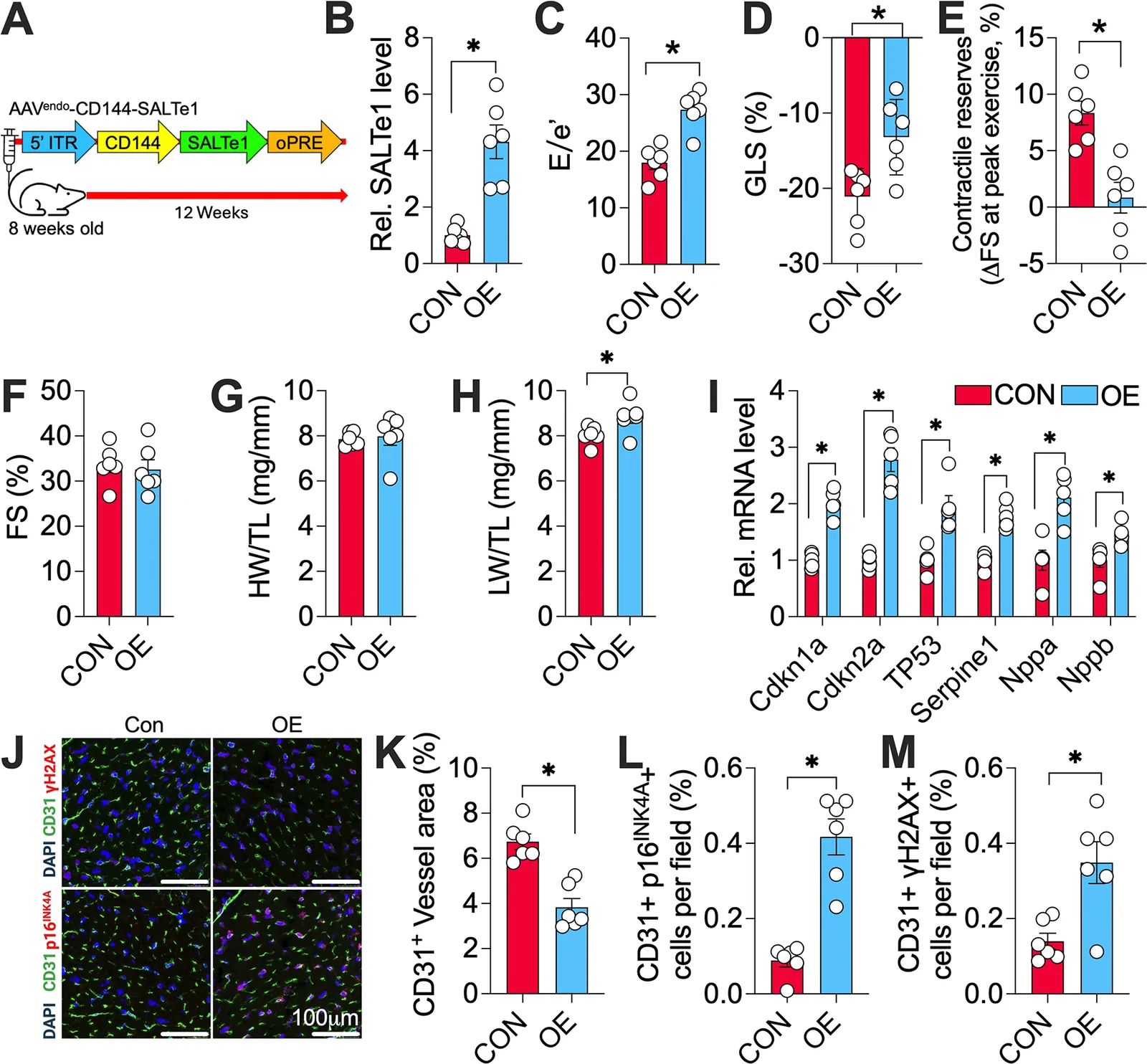
SALTe1 overexpression induces endothelial ageing and cardiac dysfunction. **A**, Schematic diagram of 8-week-old mice injected with AAV^endo^ expressing control (CON) or SALTe1 (OE). **B**, SALTe1 RNA level in the heart; *n* = 6 mice/group. **C**, Quantification of early diastolic velocity of mitral inflow (*E*) to the early diastolic velocity of mitral annular motion (e’) ratio; *n* = 6 mice/group. **D**, Global longitudinal strain (GLS); *n* = 6 mice/group. **E**, Contractile reserves; *n* = 6 mice/group. **F**, Fractional shortening (FS); *n* = 6 mice/group. **G-H**, Heart weight (HW) or lung weight (LW) to tibial length (TL); *n* = 6 mice/group. **I**, Gene expression of Cdkn2a, Cdkn1a, TP53, Serpine1, Nppa, and Nppb in the heart; *n* = 4 mice/group. **J-M**, Representative images and quantification of CD31, Cdkn2a^INK4A^ (p16^INK4A^), or γH2AX stainings; *n* = 6 mice/group. Data shown as mean ± SEM. *P < .05 by unpaired two-tailed Student’s *t*-test

**Figure 4:**
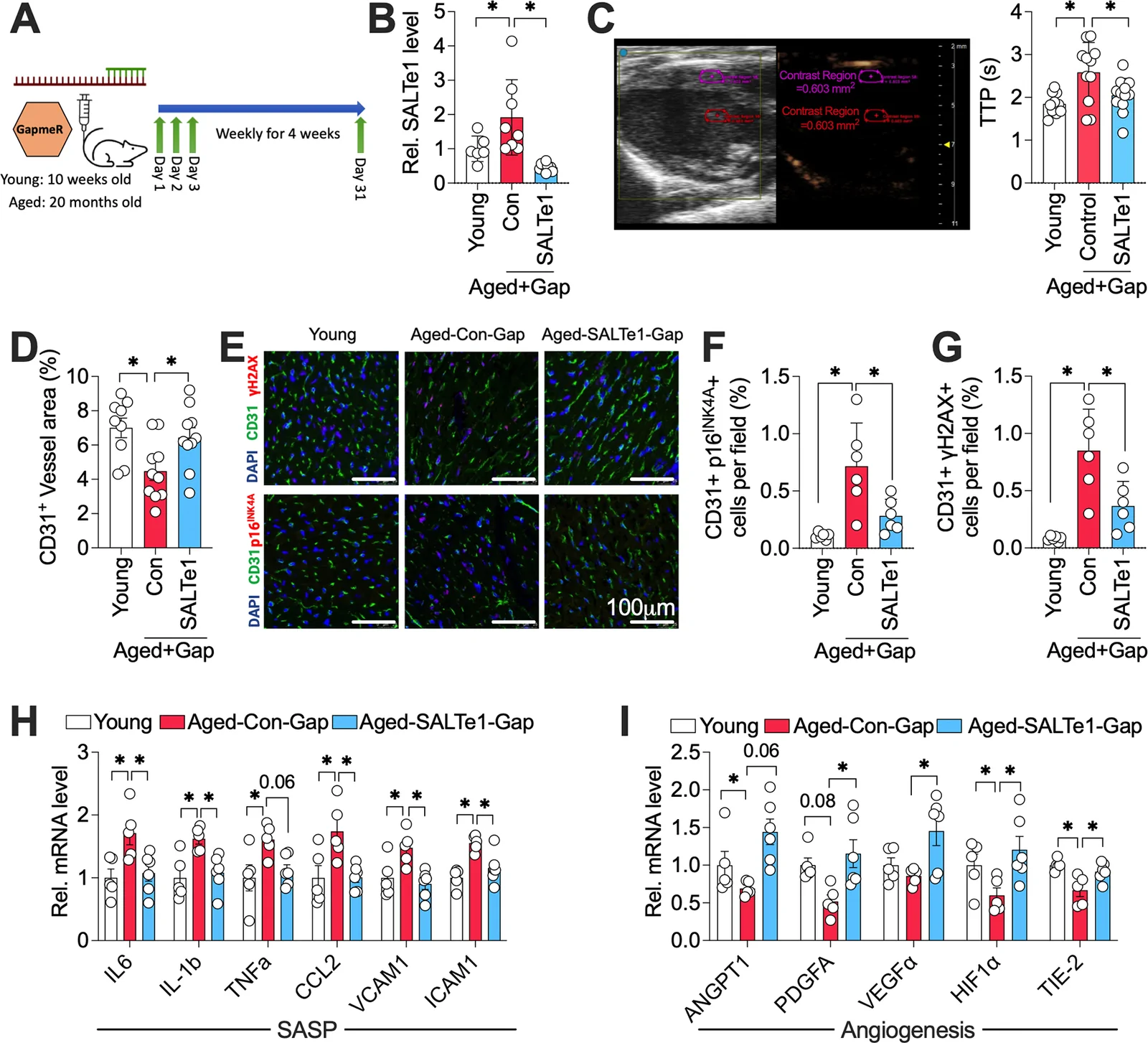
SALTe1 inhibition attenuates vascular ageing in aged hearts. **A**, Schematic diagram of young and aged mice injected with scrambled control GapmeR (control Gap) or SALTe1 specific GapmeR (SALTe1 Gap). B, SALTe1 RNA level in the hearts measured by QRT-PCR; *n* = 6–10 mice/group. **C**, Representative images and quantification of cardiac perfusion function as time to peak (T-to-P, TTP) measured by contract echocardiography; *n* = 10–12 mice/group. **D**, Quantification of CD31 positive cells; *n* = 9–10 mice/group. **E-G**, Representative images and quantification of CD31, Cdkn2a^INK4A^ (p16^INK4A^), or γH2AX stainings; *n* = 6mice/group. **H**, Gene expression of IL6, IL-1β, TNFα, CCL2, VCAM1, ICAM1; *n* = 5–6 mice/group. I, Gene expression of IL6, IL-1β, TNFα, CCL2, VCAM1, ICAM1; *n* = 5–6 mice/group. Data shown as mean ± SEM.*P < .05 by One-way ANOVA with Dunnett’s post hoc test

**Figure 5:**
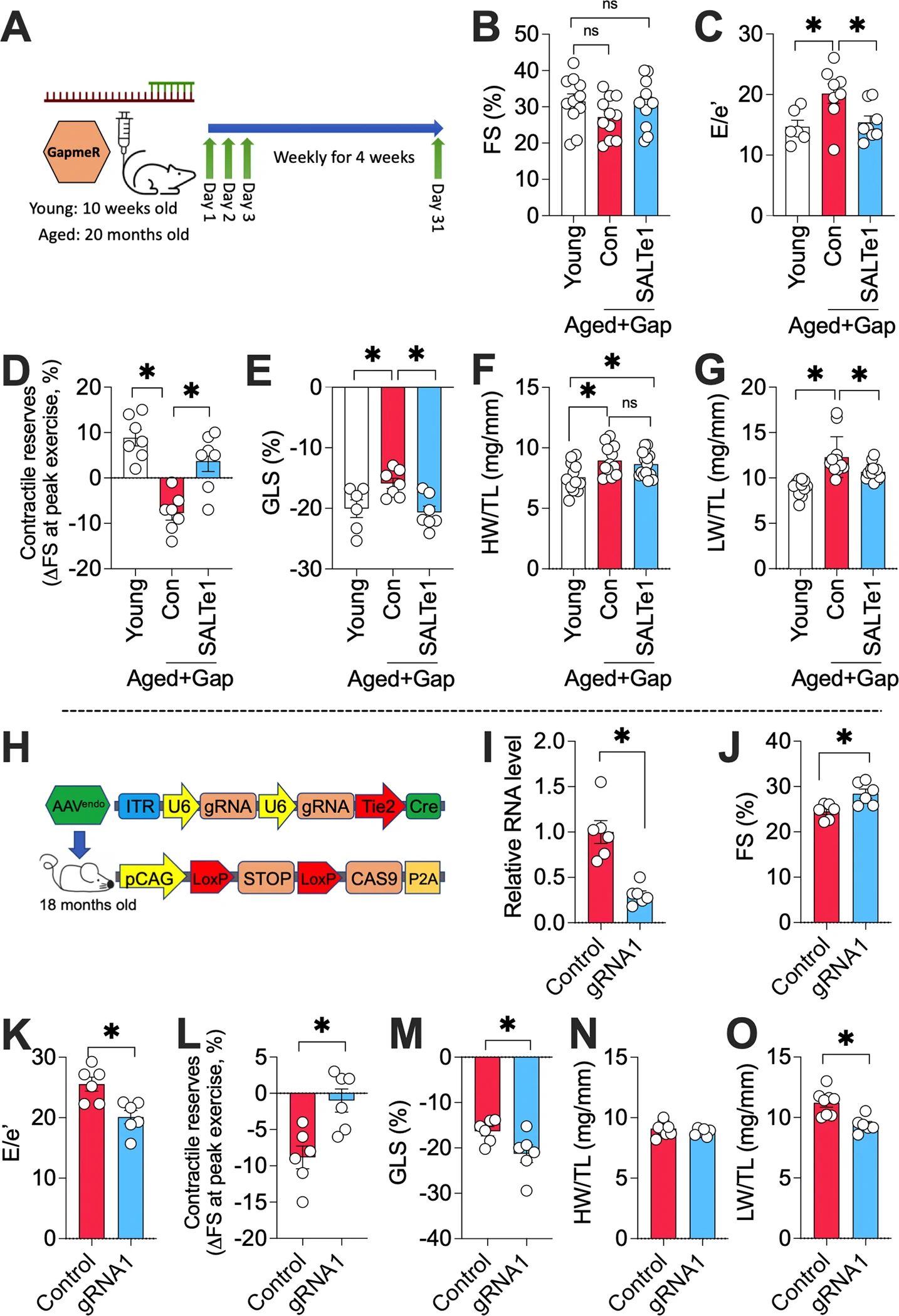
SALTe1 inhibition improves cardiac function in aged hearts. **A**, Schematic diagram of young and aged mice injected with scrambled control GapmeR (control Gap) or SALTe1 specific GapmeR (SALTe1 Gap). **B**, Fractional shortening (FS); *n* = 11–13 mice/group. **C**, Quantification of early diastolic velocity of mitral inflow (*E*) to the early diastolic velocity of mitral annular motion (e’) ratio; *n* = 6–8 mice/group. **D**, Contractile reserves; *n* = 7 mice/group. **E**, Global longitudinal strain (GLS); *n* = 6–7 mice/group. **F-G**, Heart weight (HW) or lung weight (LW) to tibial length (TL); *n* = 12–15mice/group. **H**, Schematic of experimental strategy used to knockdown SALTe1 in endothelial cells in the hearts in vivo. **I**, SALTe1 RNA level in the hearts from mice treated with AAV^endo^ expressing scrambled control (control) or gRNA1 targeting SALTe1; *n* = 6mice/group. **J**, FS; *n* = 6 mice/group. **K**, E to e’ ratio; *n* = 6 mice/group. **L**, Contractile reserves; *n* = 6 mice/group. **M**, GLS; *n* = 6 mice/group. **N**, HW/TL; *n* = 6 mice/group. **O**, LW/TL; *n* = 6 mice/group. Data shown as mean ± SEM. *P < .05 by One-way ANOVA with Dunnett’s *post hoc* test in B-G; and by unpaired two-tailed Student’s *t*-test in I-O; ns, not significant

**Figure 6:**
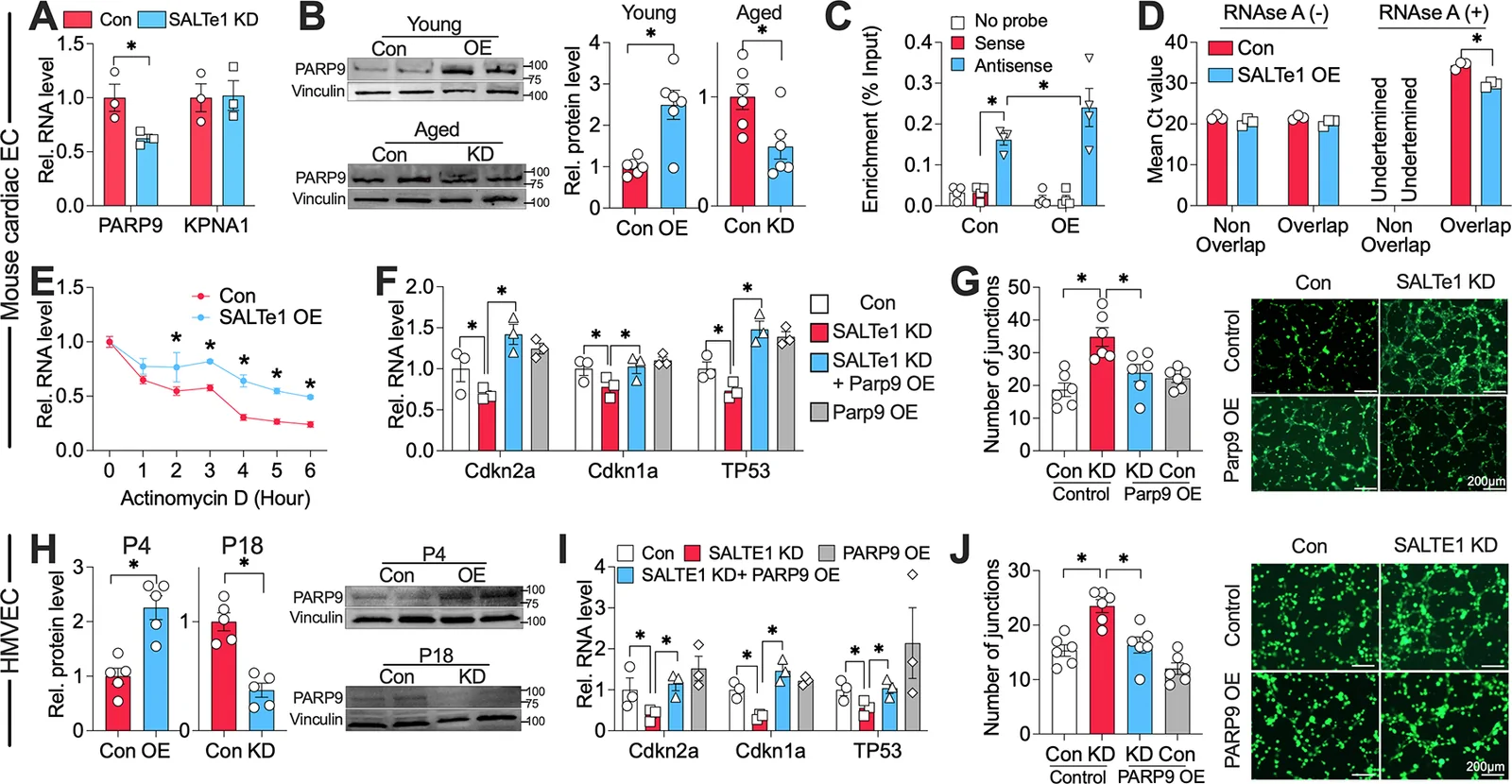
SALTe1 regulates endothelial cell function through PARP9. **A,** Gene expression of PARP9 and KPNA1 in endothelial cells (ECs) treated with scrambled control (Con) or SALTe1 GapmeR (SALTe1 KD); *n* = 3 independent biological replicates/group. **B,** Protein expression of PARP9 in ECs treated with lentivirus expressing control vector (Con) or SALTe1 (OE), or with scrambled control (Con) or SALTe1 specific GapmeR (KD); *n* = 6 independent biological replicates/group. **C,** PARP9 mRNA enrichment in RNAs extracted from ECs treated with control (Con) or lentiviral SALTe1 overexpression (OE) and pulldown using probes targeting sense or antisense SALTe1 sequence or with no probe control; *n* = 4 independent biological replicates/group. **D,** Mean Ct value from QRT-PCR in SALTe1 and PARP9 overlapping or non-overlapping area in ECs treated with (+) or without (−) RNAse A; *n* = 3 independent biological replicates/group. **E,** PARP9 RNA level in ECs treated with Actinomycin D with or without SALTe1 lentivirus (OE); *n* = 3 independent biological replicates/group. **F,** Gene expression of Cdkn2a, Cdkn1a, and TP53 in ECs treated with SALTe1 GapmeR (KD) in the presence or absence of PARP9 lentivirus (OE); *n* = 3 independent biological replicates/group. **G,** Representative images and quantification of tube formation; *n* = 6 independent biological replicates/group. **H,** PARP9 protein expression in HMVECs passaged for 4 times (P4) and 18 times (P18), and treated with control (Con) or lentiviral SALTe1 overexpression (OE), or SALTe1 GapmeR (KD); *n* = 5 independent biological replicates/group. **I,** Gene expression of Cdkn2a, Cdkn1a, and TP53 in HMVECs treated with SALTe1 GapmeR (KD) in the presence or absence of PARP9 lentivirus (OE); *n* = 3 independent biological replicates/group. **J,** Representative images and quantification of tube formation; *n* = 6 independent biological replicates/group. Data shown as mean ± SEM. *P < .05 by unpaired two-tailed Student’s *t*-test in A-B, D, E, H; by One-way ANOVA with Dunnett’s *post hoc* test in C, F, and I, and by Two-way ANOVA with Tukey’s *post hoc* test in G and J

**Figure 7:**
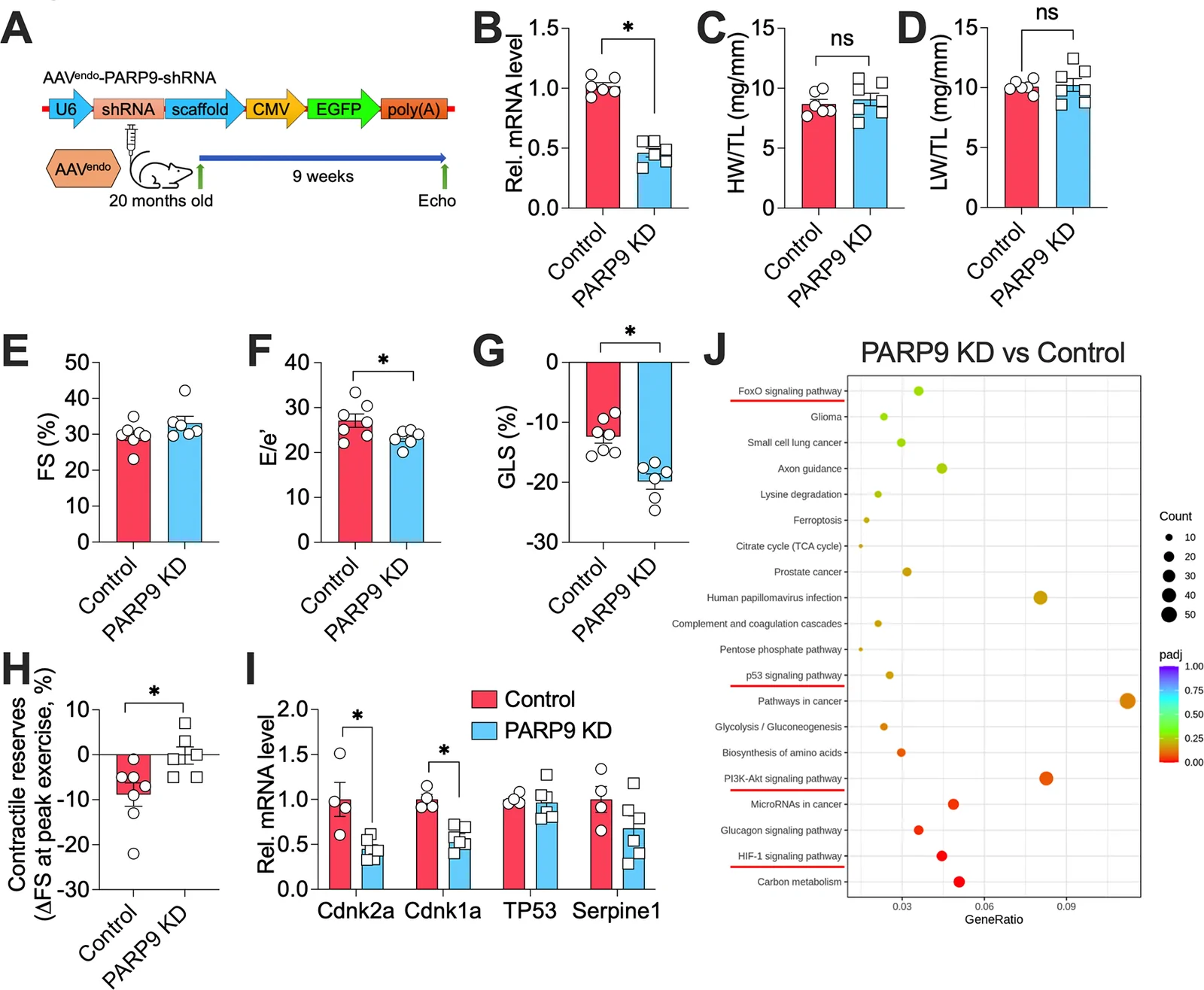
PARP9 inhibition improves cardiac function in aged hearts. **A**, Schematic of experimental strategy used to knockdown PARP9 in the heart. **B**, PARP9 gene expression in the hearts from mice injected with scramble shRNA (Control) or PARP9 specific shRNA (PARP9 KD); *n* = 6–7 mice/group. **C**, Heart weight (HW) to tibial length (TL) ratio; *n* = 6–7 mice/group. **D**, Lung weight (LW) to TL ratio; *n* = 6–7 mice/group. **E**, FS; *n* = 6–7 mice/group. **F**, E/e’; *n* = 6–7 mice/group. G GLS; *n* = 6–7 mice/group. **H**, Contractile reserves; *n* = 6–7mice/group. **I**, Gene expression of Cdkn2a, Cdkn1a, TP53, and Serpine1 in the heart; *n* = 4–6 mice/group. **J**, Top GO pathways from RNA sequencing from ECs treated with scrambled control (Control) or PARP9 siRNA (PARP9 KD). Data shown as mean ± SEM. *P < .05 by unpaired two-tailed Student’s *t*-test; ns, not significant

## Data Availability

RNA-seq raw data had been deposited to NCBI SRA (PRJNA811435) and GEO (GSE229891). The data, methods, and study materials used to conduct the research will be available from the corresponding author on reasonable request.

## Translational perspective

Ageing-related microvascular dysfunction is associated with cardiac functional decline, yet the mechanisms and potential interventions remain unclear. This study identifies the endothelial IncRNA SALTe1 as a key driver of microvascular ageing and demon-strates that its inhibition reverses vascular senescence and improves cardiac function, highlighting SALTe1 as a promising therapeutic target.
