# Supplementary material for: Long noncoding RNA SALTe1, microvascular ageing, and cardiac dysfunction
Source: Eur Heart J. Author manuscript; Available in PMC 2026 Oct 27. (PMC13711036; doi:10.1093/eurheartj/ehag356)
Supplement: Supplementary file 1 [file NIHMS2215143-supplement-Supplemnentary_material.pdf]

SUPPLEMENTARY MATERIALS

An Exercise-Responsive Long Noncoding RNA Axis Governing Microvascular Aging and Cardiac Dysfunction

Haobo Li[1,2,*], Xiao Xiao[1], Yirong Zhou[2,3,4], Yajing Zhang[1,2], Jiayi Kang[1], Zixun Yuan[5], Jiaqi He[5], Justin Guerra[5], Carolin Lerchenmüller[6,7,8,9], Kenneth B. Margulies[10], Aaron D. Aguirre[2,3,4], Jason D Roh[2,3], Anthony Rosenzweig[5,*]

*Corresponding author:

Dr. Haobo Li, PhD, Email: haobo.li@mgh.harvard.edu

Dr. Anthony Rosenzweig, MD, Email: anthros@med.umich.edu

Supplemental Materials includes:

Supplementary Materials and Methods

Supplementary Figure S1-6.

Supplementary Table S1-6.

Materials and Methods

Senolytic mouse study

The senolytic fisetin (S2298, Selleck Chemicals) was diluted in water containing 0.1% DMSO and administered by oral gavage at 20 mg/kg/day[1] for three weeks to 20-month-old mice. Ten-week- and 20-month-old mice treated with the vehicle solution (0.1% DMSO) served as controls. Cardiac function was assessed by echocardiography.

RNA sequencing

RNA sequencing was performed by the MGH NextGen Sequencing Core or Novogene. Total RNA was extracted from murine hearts using RNA prep kit (R2050, Zymo Research). Libraries were constructed from polyA-selected RNA and sequenced on an Illumina HiSeq 2500 instrument in High-Output mode as paired end 100. Raw sequence files were converted to the FASTQ format using STAR aligner. For lncRNAs identification, annotation was performed using custom noncoding RNA annotation database combining list of lncRNAs from ENSEMBL, NONCODE, and lincRNAdb. For protein coding genes, annotation was performed using EnsEMBL v96 reference (mm39/GRCm39). R package DESeq2 was used for gene expression analysis. Genes were considered differentially expressed if fold-change >1.5 or < -1.5 with *p* value <0.05. RNA-seq raw data had been deposited to NCBI Sequence Read Archive (SRA) with accession# PRJNA811435 (exercised aged hearts), and Gene Expression Omnibus (GEO) with accession# GSE229891 (endothelial cells with PARP9 knockdown).

Locked nucleic acid-GapmeR injection *in vivo*

Locked nucleic acid (LNA) treatment was performed as described[2-4]. Briefly, 20-month-old C57BL/6J male mice were injected subcutaneously with 25 mg/kg of LNA-GapmeR (Qiagen) targeting SALTe1 (LNA-GapmeR-SALTe1, 5'-A*T*C*T*A*T*G*G*T*G*C*T*C*G*T*T-3') or scrambled control (LNA-Control-GapmeR, 5'-A*A*C*A*C*G*T*C*T*A*T*A*C*G*C-3') (*indicated an LNA modification) reconstituted in saline. Mice were injected for three consecutive days followed by weekly injection throughout the experiments.

Echocardiographic studies

Echocardiography was performed using a Vevo 3100 system (FUJIFILM VisualSonics) with an

MX250S high-frequency transducer[5]. Mice were anesthetized with 5% isoflurane for induction and 1.0–1.5% for maintenance, with body temperature controlled to maintain heart rates between 425 and 500 beats per minute. Left ventricular (LV) systolic function was assessed by M-mode echocardiography from parasternal short-axis views at the level of the papillary muscles. Fractional shortening (FS) was calculated as FS (%) = [(LVIDd − LVIDs) / LVIDd] × 100. Diastolic function was evaluated in apical four-chamber views using pulsed-wave and tissue Doppler imaging at the mitral valve, including early (E) and late (A) mitral inflow velocities, early diastolic mitral annular velocity (e′), and deceleration time. LV wall thicknesses and internal diameters at end-diastole and end-systole were measured from M-mode images. Images were analyzed using Vevo LAB software (v5.10.0). All measurements were averaged from at least three cardiac cycles, and investigators were blinded to group allocation.

Stress echocardiography was used to assess exercise capacity and cardiac reserves in mice as described[6]. Resting parasternal short-axis M-mode images were acquired in unanesthetized mice using a Vivid E90 system (GE Healthcare) with an L8-18i-D transducer[7,8]. Resting echocardiography was performed on a separate day to avoid confounding effects on exercise testing. After three days of treadmill acclimatization, mice underwent an exhaustion test on a 45° incline treadmill (Exer 3/6, Columbus Instruments). Following a 5-min warm-up (5-15 m/min), speed was increased by 2 m/min every 2 min until exhaustion, defined as the inability to maintain pace for 3 s on three consecutive occasions. Running time and distance were recorded. Parasternal M-mode images were acquired within 30s of peak exercise and analyzed using EchoPACS (version 201, GE Healthcare). At least three measurements were averaged per mouse. Chronotropic reserve was assessed from peak heart rate (average of nine beats), and contractile reserve was determined by FS augmentation from rest to peak exercise.

***In vivo* myocardial contrast echocardiography**

Myocardial contrast echocardiography was performed using a VisualSonics Vevo 3100 system equipped with an MX250S transducer (FUJIFILM VisualSonics). After being anesthetized with 3% isoflurane, mice were fixed in a supine position on an imaging platform at 37°C. Simultaneous ECG and respiratory waveforms were acquired. Isoflurane was maintained at 1.5%–2% during the examination to obtain constant and comparable heart rates without evident cardiac depression. All animals were

scanned in parasternal long- and short-axis views, and myocardial contrast echocardiography scans were performed in nonlinear contrast mode using the amplitude modulation technique. B-mode and contrast images were displayed side by side to facilitate subsequent regional analysis. All images were acquired at a transmission frequency of 21 MHz, 10% power, a dynamic range of 40 dB, and a contrast gain of 30 dB. Microbubble contrast agent (VS-11913, FUJIFILM VisualSonics) was freshly prepared and diluted to 1:5 for each dose. The diluted agent was then constantly injected via the tail vein as a bolus of 50μL (equivalent to a dose of $2x10^7$ microbubbles). The inflow and outflow of the agent can be tracked in the imaging plane over time and plotted as a Time-Intensity curve, with the linear data used to generate the ultrasound image. Approximately 300 frames of baseline (pre-contrast) were recorded, followed by injection of the contrast agent. The Bolus phase was maintained for approximately 1,000 frames; then the burst/replenishment mode was triggered manually to rapidly destruct all microbubbles (mechanical index >0.7). The image series was then terminated approximately 1,000 frames after the burst. A two-dimensional polygonal region of interest (ROI) encompassing the anterior wall of LV was used over time to quantify contrast intensity. ROI was positioned at the midpoint of the myocardial wall to avoid epicardial vessels and rib shadows. Time-intensity analysis was then performed by VevoLAB (FUJIFILM VisualSonics). Smooth curves of time-intensity including both bolus and replenishment phases were created by averaging 32 frames. Time-to-peak-intensity and peak intensity of bolus and replenishment phases were computed to compare myocardial perfusion between mice.

Quantitative real-time polymerase chain reaction

Total RNA was isolated using RNA prep kit (R2050, Zymo Research). Reverse transcription was performed using the High-Capacity cDNA Reverse Transcription Kit (4374967, Applied Biosystems). Polymerase chain reaction (PCR) reactions were performed using SYBR Green Universal Master Mix (4334973, Applied Biosystems) and standard amplification protocols. Gene expression was normalized to RPS18 or GAPDH and calculated using the $\Delta\Delta C_t$ method. Primer sequences are listed in **Supplementary Table S6**.

Endothelial cell isolation, culture, transfection, and treatments

Human cardiac microvascular endothelial cells (HMVECs) were purchased from Lonza (CC-7030)

and verified for negativity for mycoplasma contamination. Endothelial cell (EC) identity was authenticated by using immunostaining, flow cytometry, and the expression of CD144, CD31, and eNOS mRNA. HMVECs at passages 3-5 were cultured in HMVEC growth medium supplemented with growth factors, heparin sodium under standard cell culture conditions (humidified atmosphere, 5% CO_2, 37 °C), following with SALTe1 or PARP9 knockdown or overexpression as described below.

Primary mouse cardiac ECs were prepared as described[9]. ECs were plated in 60 mm dishes at $1x10^6$ cells *per* well and cultured in EC culture medium with kits (M1168, Cell Biologics) for 24 hours. Before treatment, ECs were synchronized and cultured in EC basal medium containing 0.2% FBS. Twenty-four hours after plating, cells were transfected with negative control (25 nM; LG00000002-DDA, Qiagen) or LNA-GapmeR (25 nM; LG00247145-DDA for Gap #1; LG00781257-DDA for Gap #2; LG00781260-DDA for Gap #3, Qiagen); or scramble control (25 nM; Assay#: 129351000, Invitrogen) or PARP9 siRNA (25 nM; Assay#: s95940, Invitrogen), or TLR4 siRNA (25 nM; Assay#: 188775, Invitrogen), or Akt1 siRNA (25 nM; Assay#: 162426, Invitrogen), or ErbB2 siRNA (25 nM; Assay#: 49332, Invitrogen), ErbB3 siRNA (25 nM; Assay#: 198317, Invitrogen), or ErbB4 siRNA (25 nM; Assay#: 191926, Invitrogen) for forty-eight hours, using Lipofectamine RNAiMAX (13778150, Invitrogen) according to the manufacturer's instruction. After overnight serum starvation, ECs were treated with 50ng/mL NRG1 (5898-NR-050, R&D System), 100ng/mL IGF-1 (291-G1-01M; R&D Systems), 10nmol/L ET-1 (1160/100U, Fisher Scientific), 200μM H_2O_2 (CAS 7722-84-1, Sigma-Aldrich), 20μg/mL LPS (L2630-25MG, Sigma-Aldrich), 50ng/mL TNFα (300-01A, PeproTech), 2mM doxorubicin (D1515-10MG, Sigma-Aldrich). HMVECs were synchronized and transfected using the same protocol and conditions, with negative control (25 nM; LG00000002-DDA, Qiagen) or LNA-GapmeR for human SALTE1 (25 nM; 5'-G*T*T*A*G*C*A*A*G*T*G*A*A*G*A*G-3', LG00809797-DDA, Qiagen).

Isolation of cytosolic and nuclear fractions

Mouse ECs or HMVECs were suspended and washed with PBS and harvested for isolation of cytosolic and nuclear fractions according to the manufacturer's protocol as described in PARIS kit (Life Technologies) followed by RNA extraction and QRT-PCR as described[3]. Beta-actin or GAPDH was used as cytosolic marker and U6 was used as nuclear marker. Previously described lncRNA HOTAIR[61],

predominantly expressed in the nucleus, was used as positive control.

Lentivirus generation and transfection

The full-length mouse or human PARP9 open reading frame or SALTe1 was cloned and inserted into a modified pLVX-Tight-Puro vector (TER promoter was replaced by EF1a promoter from pLVX-Tight-Puro) at *BclI* and *MluI* sites for generating mouse or human PARP9 overexpression lentivirus vector. Lentiviral constructs were transfected into the HEK293T cells along with lentiviral packaging plasmids psPAX2 and VSVG using Lipofectamine 3000 (L3000015, Invitrogen). Virus-containing supernatant was collected, and cellular debris was removed by syringe filtration (0.22 μm pore size; Millipore). Filtered supernatant was concentrated using lenti-X Concentrator (631231, Clontech). The viral pellet was resuspended in serum-free DMEM. For infection in ECs, 24 hours after plating, ECs were incubated with DMEM medium containing lentivirus and polybrene (6 μg/ml, Millipore) for 48 hours.

CRISPR/dCAS9 and sgRNA construct design

CRISPR/dCAS9 system was used to transcriptional activation of SALTe1, 2, and 9 in ECs *in vitro* as described[3], single guide RNAs (gRNAs) targeting the SALTes promoter were designed using the Intergrated DNA Technologies (IDT) crRNA design tool and analyzed for specificity (https://www.idtdna.com/site/order/designtool/index/CRISPR_CUSTOM). Two gRNAs targeting the promoter of each SALTe and one gRNA not targeting mouse genome (as control), sequences of gRNAs were listed in **Supplementary Table S6**. gRNA was inserted into the lentiGuide-Puro vector (52963, Addgene)[10] at the *BsmBI* site. lentiGuide-Puro vector and lenti-dCAS-VP64_Blast vector (61425, Addgene)[11] were packaged as lentivirus as described above. LentiGudie-Puro lentivirus and lenti-dCAS-VP64_Blast lentivirus were infected into ECs for 72 hours to activate SALTes transcription.

Senescence β-Galactosidase Staining

ECs were fixed in 1% paraformaldehyde, processed for β-Galactosidase staining using the Senescence β-Galactosidase Staining Kit (#9860, Cell Signaling Technology) according to the manufacturer's instructions.

Telomere length quantification

Total genomic DNA was extracted from ECs after treatments using a Genomic DNA Purification

Kit (51304, Qiagen). The average telomere length was measured and calculated using the Absolute Mouse Telomere Length Quantification qPCR Kit (M8918, ScienCell) according to the manufacturer's protocol.

Ribonuclease protection assay

To determine the formation of RNA duplex between SALTe1 and Parp9, ribonuclease protection assay (RPA) was performed followed by qPCR analysis. Total RNA was extracted from ECs after indicated treatments. RNAs were then digested by DNaseI to remove genomic DNA contamination followed by RNase A (12091-021, Applied Biosystems) to remove single-stranded RNAs. RNA samples were then processed to cDNA synthesis and qPCR as described above using primers listed in **Supplementary Table S6**.

RNA pulldown

RNA pulldown was performed as previously described[12]. Briefly, a pool of ten 3′-end–labeled DNA oligonucleotide probes targeting SALTe1 was synthesized (IDT, **Supplemental Table S6**). Forty-eight hours after infection with SALTe1-overexpressing lentivirus, ECs were rinsed with PBS, fixed with 1% paraformaldehyde, and cross-linked for 10 min at room temperature with agitation, followed by quenching with 1/10 volume of glycine. Cells were washed twice with PBS, collected, and lysed in buffer containing 50 mM Tris-HCl (pH 7.0), 10 mM EDTA, and 1% SDS, supplemented with RNase inhibitor (200 U/mL; Invitrogen, 10777019) and protease inhibitor cocktail (5 µL/mL; Thermo Scientific, 78442). Lysates were then subjected to serial sonication. The supernatants were hybridized with biotinylated probes for 8 h at room temperature with moderate agitation, followed by overnight incubation with magnetic streptavidin beads (Invitrogen) supplemented with RNase inhibitor and protease inhibitors. Beads were collected using a magnetic stand, washed five times with wash buffer (0.5% SDS, 2× SSC), and digested with proteinase K for 45min at 50°C, followed by 10min at 95°C. Beads were then removed, and the supernatant was processed for RNA extraction followed by RT-qPCR of Parp9 mRNA expression using a standard protocol described above.

RNA stability assay

ECs were isolated and seeded into 6-well plate for 24 hours followed by lentivirus overexpression

of SALTe1 or control for 48 hours. ECs were then treated with actinomycin D (5 μg/ml, Sigma-Aldrich) for 0, 1, 2, 3, 4, 5, and 6 hours to inhibit transcription. ECs were then harvested for RNA extraction followed by RT-qPCR of Parp9 mRNA expression. Fold changes of ΔCt from each time point and time point 0 hour of Actinomycin D treatment were calculated and non-linear regression curves using GraphPad Prism to determine transcript stability over time.

Tube formation assay

96-well plates were pre-coated with 50 μl LDEV-free Matrigel Basement Membrane Matrix at 37°C for one hour. ECs were then seeded onto the coated plates at a density of $2x10^4$ cell/well and incubated in a humidified incubator for 6 hours. Calcein AM (17783, Invitrogen) were added at a final concentration of 6 μM to each well for 30min to label the cells. Calcein AM-labelled cells were imaged immediately using a Leica Confocal TCS SP8 Microscope.

Cell migration assay

ECs migration was measured using Cell Migration Kit (CBA-100-FN, Cell Biolabs) according to the manufacturer's instructions. ECs in 300 μl serum-free medium were seeded onto the upper chamber of the inserts at a density of $1.0x10^6$ cells/ml per well. The lower chamber was added with 500 μl complete medium. After 8 hours, medium from the inside of the insert were aspirated. Non-migratory ECs on the interior of the insert were carefully removed with a cotton swab. The migrated ECs on the exterior of the insert were then stained with 0.1% crystal violet at room temperature for 10min and imaged using a Leica Confocal TCS SP8 Microscope.

Protein coding potential

Transcripts of SALTe1 full cDNA sequence and one small ORF (sORF) were synthesized by IDT. These transcripts were cloned upstream of a p3xFLAG-CMV-13 expression vector (E4776, Sigma) using EcoRI and BamHI restriction sites. HEK293T cells were transfected with 2,000 ng plasmid using Lipofectamine 3000 (L3000015, Invitrogen) and protein lysate was isolated 72 hours after transfection, followed by immunoblotting for FLAG Tag (1:2000, 8146S, Cell Signaling Technology). p3xFLAG-CMV-13 expression plasmid (E4776, Sigma) was used as positive control.

RNA-binding protein immunoprecipitation was performed using Magna RIP RNA-Binding Protein

Immunoprecipitation Kit (17-701, Millipore Sigma) as described[3]. Briefly, ECs were rinsed with PBS, fixed with 1% paraformaldehyde, and cross-linked under agitation for 10 min at room temperature, then quenched by adding 1/10 volume of glycine. After two PBS washes, cells were collected and lysated with complete RIP lysis buffer (with protease inhibitor cocktail and RNase inhibitor). Supernatants were incubated with RPL4 antibody overnight under moderate agitation at 4°C. Beads were then collected using a magnetic stand and washed five times with wash buffer. Beads were then processed to RNA extraction using standard protocol followed by QRT-PCR.

Generation and injection of adeno-associated virus

AAV vector with cardiomyocyte-specific troponin I promoter was provided by the Seidman Lab[13] and promoter has been modified to the EC-specific promoter CD144 (for SALTe1) or U6 (for shRNA). AAV-PARP9 shRNA was constructed by subcloning the mouse PARP9-specific shRNA (5'-ACCGCAAG CAAATGTTCGGTAATTTCTCGAGAAATTACCGAACATTTGCTTG-3'; scrambled control: 5'-CCGGCC TAAGGTTAAGTCGCCCTCGCTCGAGCGAGGGCGACTTAACCTTAGGTTTTTG-3'), or AAV-SALTe1 was constructed by subcloning the mouse SALTe1 (AAV-GFP as control) into the above-mentioned modified AAV vector at *NcoI* and *BsrGI* sites. Recombinant AAV-Control and AAV-PARP9 shRNA or AAV-SALTe1 were produced using a triple transfection approach with a modified capsid by inserting AEGDVAR peptide to AAV2, which previously shown to have high efficacy and specificity in transducing cardiac ECs *in vivo* through directed evolution capsid screening[14], in HEK293T cells in 15cm dishes with polyethylenimine and were harvested after 60 hours incubation, purified by filtration and iodixanol gradient ultracentrifugation, and quantified as described[15]. A total amount of $2*10^{12}$ GC/mouse of AAV (AAV-control, AAV-SALTe1, or AAV-PARP9 shRNA) was delivered by tail vein injection to mice at the indicated age. For AAV mediated SALTe1 gene depletion in CAS9-knockin mice, two sets of gRNAs targeting either SALTe1 exon1 (5'-AGGGAAGCTATATTGATGTG-3; 5'-TGAGAGGGAAGCTATATTGA TGTGAGGGGG -3') or exon 3 (5'-GGGCATCAGATACTACAACC-3'; 5'- AAGAGGGCATCAGATACTAC AACCTGGCC A-3') using the GPP Web Portal (Broad Institute). The gRNA sequences were synthesized as single-stranded oligos, annealed, and inserted into AAV-U6gRNA-U6gRNA-Tie2-Cre plasmids (promoter has been modified to Tie2 based on Addgene #87682)[16] at SapI and NheI sites. AAV was

produced as described above. $2*10^{12}$ GC/mouse AAV was injected into the $Rosa^{Cas9GFP/Cas9GFP}$ mice at the indicated age via tail vein.

Histology and immunohistochemistry

Mid-ventricular sections immunofluorescent double staining was performed. Anti-CD31 PE-conjugated antibody (FAB3628P-100, Novus Biologicals) was incubated at 4°C overnight was used for EC identification. Cellular senescence markers $Cdkn2a^{INK4A}$ (PA5-20379, Thermo Scientific) or γH2AX (9718S, Cell Signaling Technology), and Alexa Fluor 594 goat anti-rat IgG secondary antibody (A-11007, Thermo Fisher Scientific) were used. Nuclei were counterstained with DAPI (D21490, Thermo Fisher Scientific) and the slides were imaged on a Leica Confocal TCS SP8 Microscope. CD31 and $Cdkn2a^{INK4A}$ or γH2AX double positive ECs (~250 cells/heart) was quantified from six randomly selected sections per heart using ImageJ (NIH). Image analysis was blinded.

Immunoblotting

Protein was isolated using radioimmunoprecipitation assay (RIPA) buffer and processed using standard Western blotting protocols. Image quantification was performed with the ChemiDoc system (Bio-Rad). The following primary antibodies were used: rabbit-anti-mouse RPL4 (1:2000; 11302-1-AP, Proteintech), rabbit-anti-mouse PARP9 (1:2000; PA5-48254, Thermo Fisher Scientific), rabbit-anti-mouse Vinculin (1:3000; V9264-200UL, Sigma-Aldrich).

Supplementary Figure S1

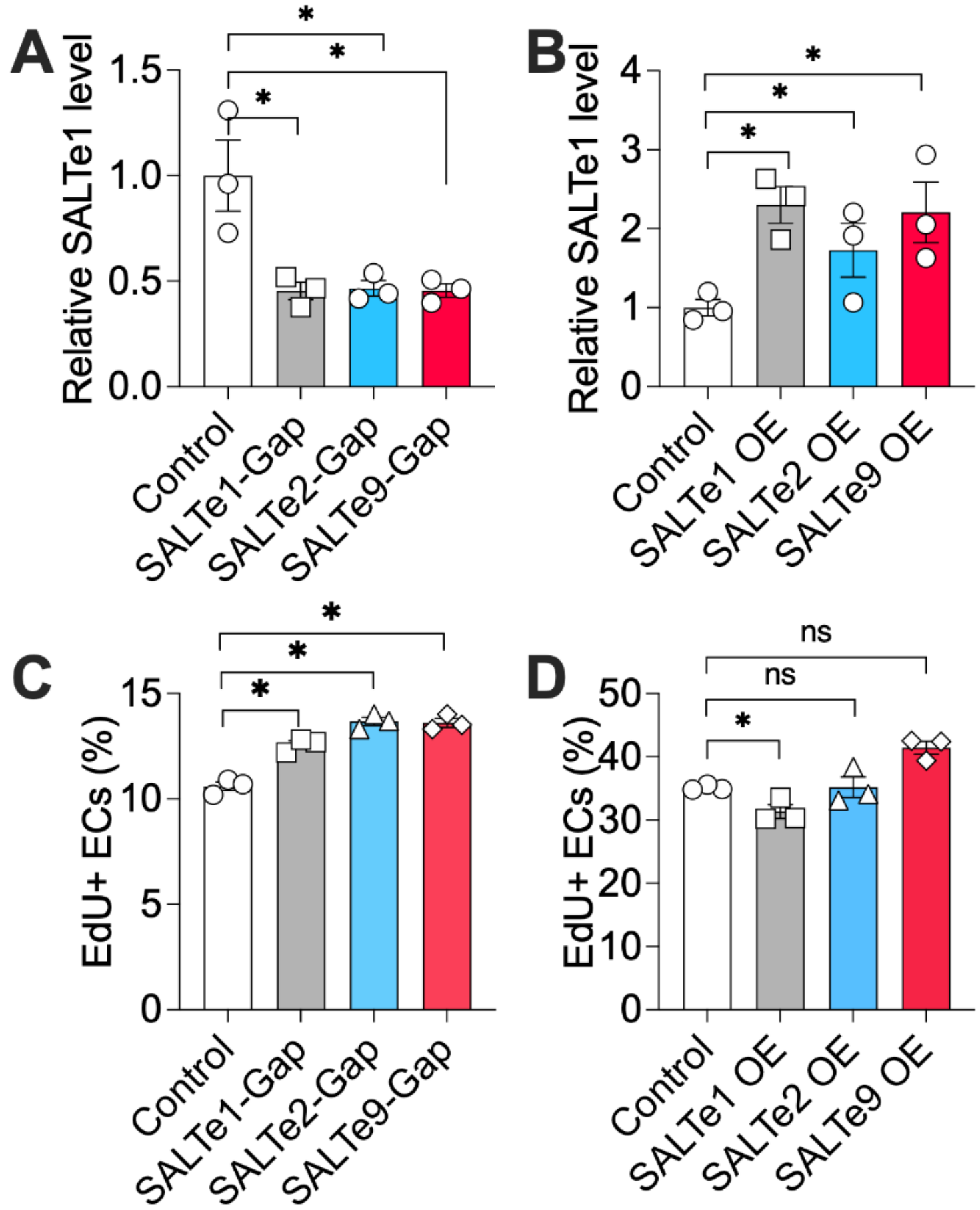

Supplementary Figure S1. SALTe1 regulates endothelial cells proliferation. A, SALTe1 RNA level in endothelial cells (ECs) treated with scrambled control (control) or SALTe1, 2, and 9 specific GapmeRs (Gap). **B,** Quantification of EdU positive ECs treated with scrambled control or SALTe1, 2, and 9 specific GapmeRs (Gap). **C,** SALTe1 RNA level in ECs treated with control vector (control) or SALTe1, 2, and 9 lentiviruses (OE). **D,** Quantification of EdU positive ECs treated with control vector (control) or SALTe1, 2, and 9 lentiviruses (OE). Data shown as mean ± SEM. A-D: n=3 independent biological replicates/group. *p<0.05 by One-way ANOVA with Dunnett's post hoc test.

Supplementary Figure S2

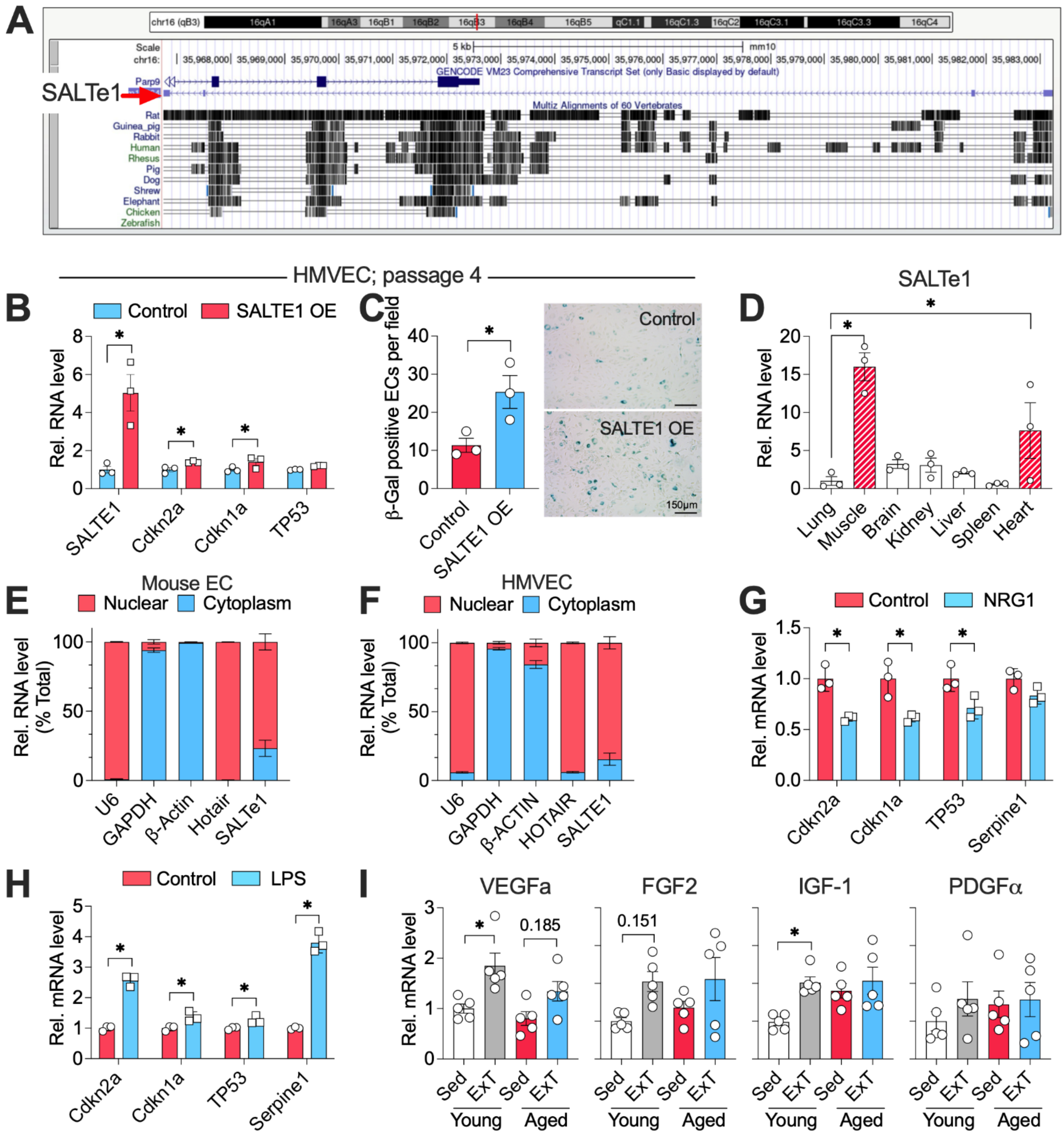

Supplementary Figure S2. SALTe1's expression in different organs and cellular fractions. A, SALTe1 sequence alignment in multiple species. **B,** Gene expression of SALTe1, Cdkn2a, Cdkn1a, and TP53 in HMVECs treated with lentivirus expressing empty vector control (control) or human SALTE1 (SALTE1 OE); n=3 independent biological replicates/group. **C,** Quantification and representative images

of β-galactosidase (β-gal) staining; n=3 independent biological replicates/group. **D,** SALTe1 RNA levels in different organs; n=3 mice/group. **E,** SALTe1, Hotair, β-Actin, GAPDH, and U6 RNA levels in nuclear and cytoplasmic fractions from mouse cardiac ECs; n=3 independent biological replicates/group. **F,** SALTE1, HOTAIR, β-ACTIN, GAPDH, and U6 RNA levels in nuclear and cytoplasmic fractions from HMVECs; n=3 independent biological replicates/group. **G,** Gene expression of Cdkn2a, Cdkn1a, TP53, and Serpine1 in ECs treated with DMSO (control) or NRG1; n=3 independent biological replicates/group. **H,** Gene expression of Cdkn2a, Cdkn1a, TP53, RB1, and Serpine1 in ECs treated with DMSO (control) or LPS; n=3 independent biological replicates/group. **I,** Gene expression of NRG1, VEGFa, IGF-1, FGF2, and PDGFα in hearts from young (8-week-old) and aged (20-month-old) mice with sedentary (Sed) or eight weeks of voluntary wheel running (ExT); n=5 mice/group. Data shown as mean ± SEM. *p<0.05 by unpaired two-tailed Student's t-test in B-C, E-H, by One-way ANOVA with Dunnett's post hoc test in D, and by Two-way ANOVA with Tukey's post hoc test in I.

Supplementary Figure S3

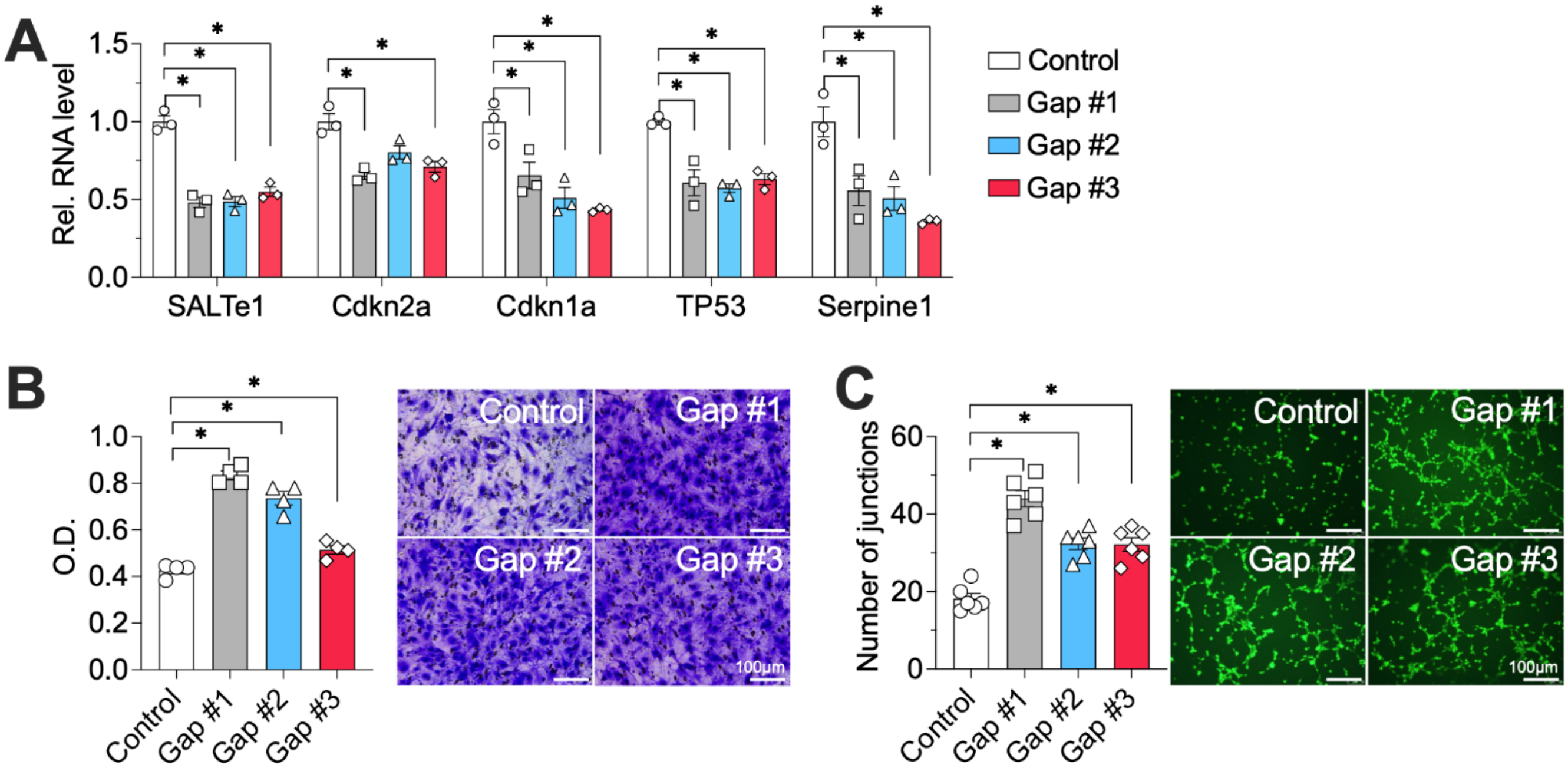

Supplementary Figure S3. SALTe1 inhibition reduces senescence and improves angiogenesis. A, Gene expression of SALTe1, Cdkn2a, Cdkn1a, TP53, and Serpine1 in ECs treated with Control, GapmeR #1 (Gap #1), GapmeR #2 (Gap #2), or GapmeR #3 (Gap #3). **B,** Representative images and quantification of cell migration in ECs treated with control, Gap #1, #2, and #3. **C,** Representative images and quantification of tube formation. Data shown as mean ± SEM. A-C: n=3 independent biological replicates/group. *p<0.05 by One-way ANOVA with Dunnett's post hoc test.

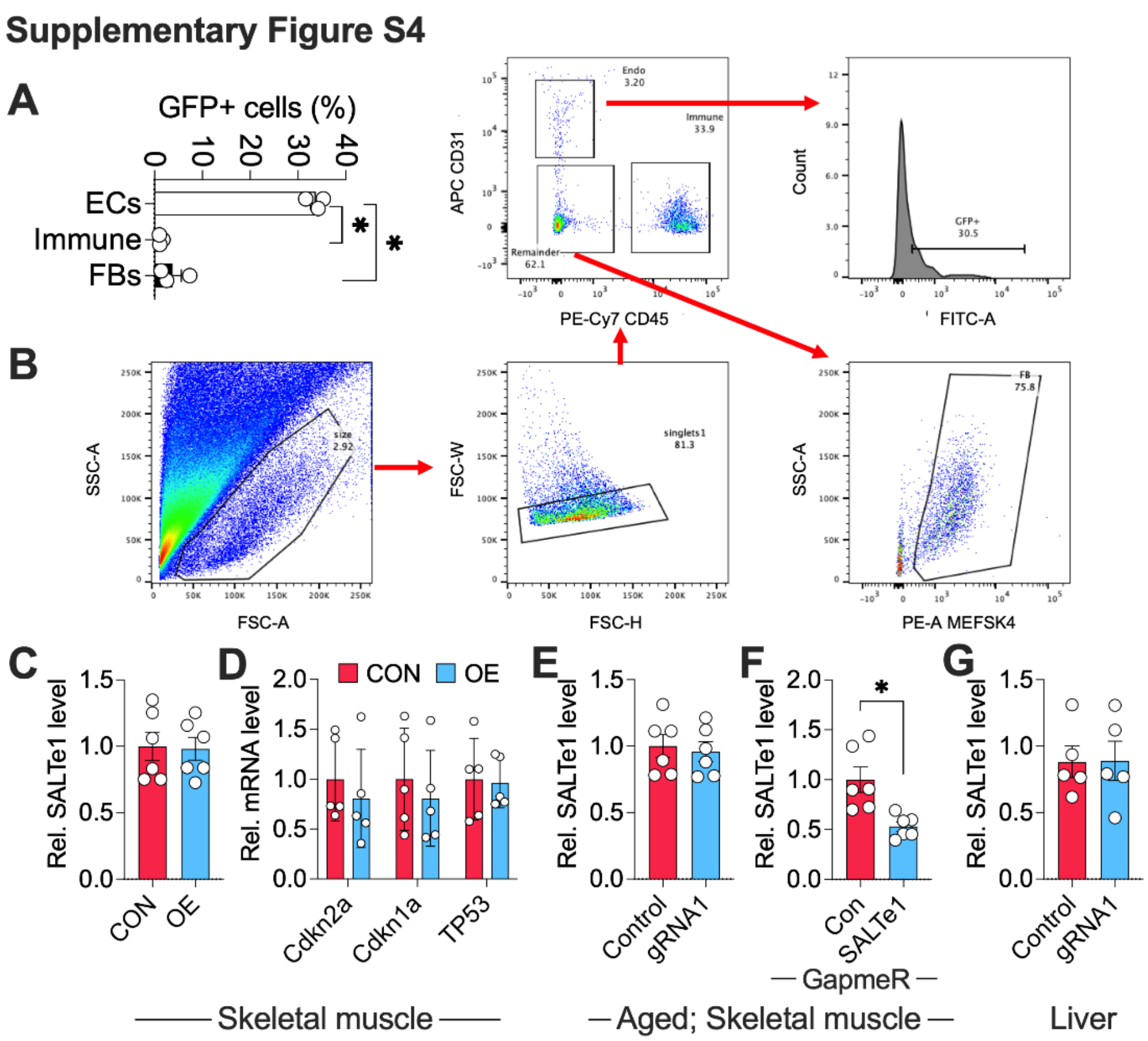

Supplementary Figure S4. SALTe1 expression in the skeletal muscle in mice with AAV^{endo} or GapmeR. A, Percentage of GFP-positive cells in ECs (CD31-positive and CD45-negative), immune cells (CD45-positive), and fibroblasts (FBs, MEFSK4-positive) isolated from hearts four weeks after injection of AAV^{endo}-GFP; n=3 mice/group. **B,** Flow cytometry gating strategy. **C,** SALTe1 expression in the skeletal muscle in 8-week-old mice injected with AAV^{endo}-Control (CON) or AAV^{endo}-SALTe1 (OE); n=6 mice/group. **D,** Gene expression of SALTe1, Cdkn2a, Cdkn1a, TP53, and Serpine1 in the skeletal muscle in 8-week-old mice injected with AAV^{endo}-Control (CON) or AAV^{endo}-SALTe1 (OE); n=5 mice/group. **E,** SALTe1 RNA expression in the skeletal muscle in 18-month-old CAS9 knockin mice injected with AAV^{endo}-Control (Control) or AAV^{endo}-gRNA1 (gRNA1); n=6 mice/group. **F,** SALTe1 RNA expression in the skeletal muscle in 20-month-old mice injected with GapmeR-Control (Gap-Con) or GapmeR-SALTe1 (Gap-SALTe1); n=6 mice/group. **G,** SALTe1 RNA expression in the liver in 18-month-old CAS9 knockin mice injected with AAV^{endo}-Control (Control) or AAV^{endo}-gRNA1 (gRNA1); n=5 mice/group. Data shown as mean ± SEM. *$p<0.05$ by One-way ANOVA with Dunnett's post hoc test in A; by unpaired two-tailed Student's t-test in C-G.

Supplementary Figure S5

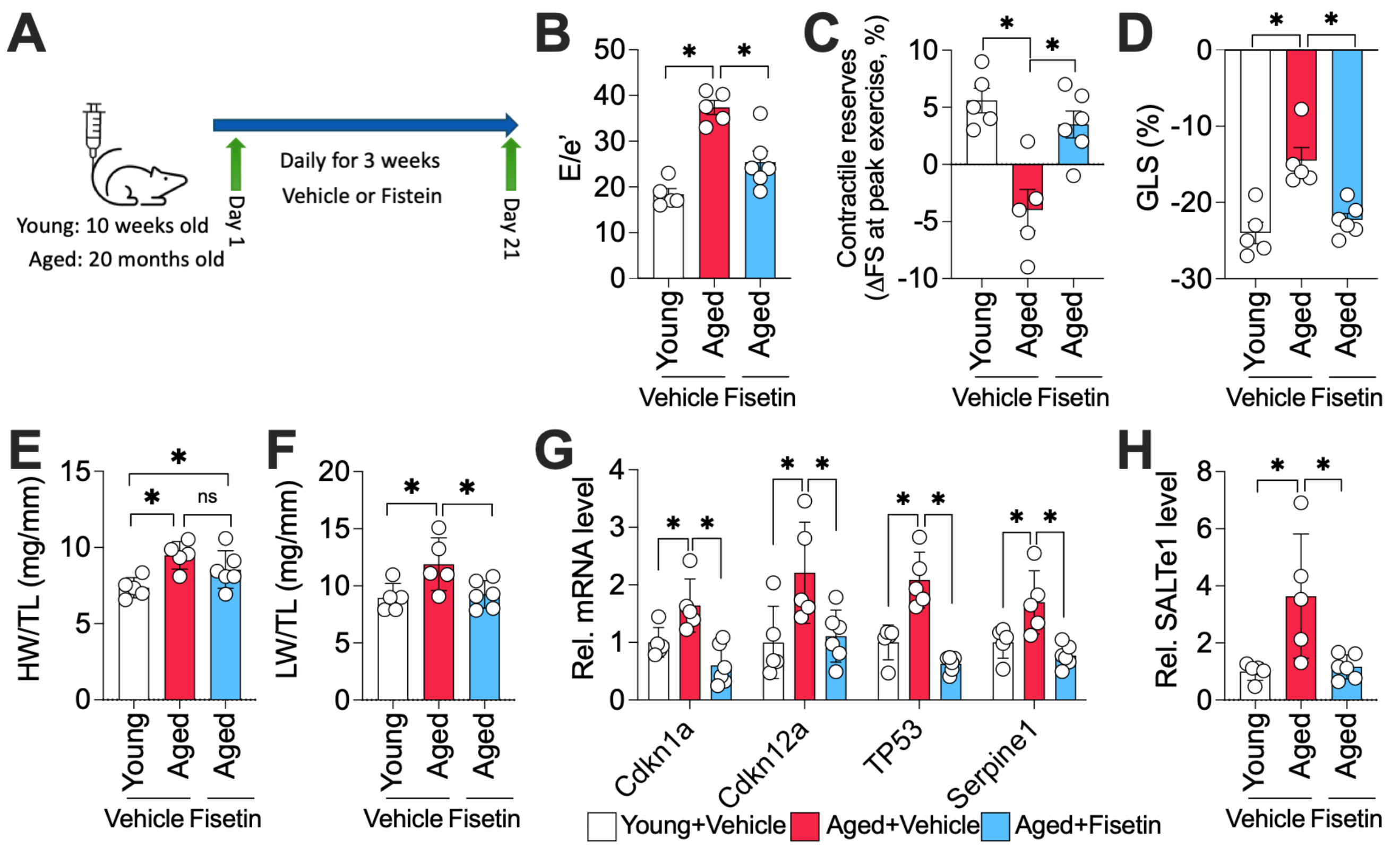

Supplementary Figure S5. Senolytic fisetin attenuates senescence and improves cardiac function in aged mice. A, Schematic diagram of 10-week and 20-week-old mice treated with vehicle or fisetin. **B,** Quantification of early diastolic velocity of mitral inflow (E) to the early diastolic velocity of mitral annular motion (e') ratio. **C,** Contractile reserves. **D,** Global longitudinal strain (GLS). **E-F**, Heart weight (HW) or lung weight (LW) to tibial length (TL). **G,** Gene expression of Cdkn2a, Cdkn1a, TP53, Serpine1 in the heart. **H,** SALTe1 RNA levels in the heart. Data shown as mean ± SEM. B-H: n=5-6 mice/group. *p<0.05 by One-way ANOVA with Dunnett's post hoc test.

Supplementary Figure S6

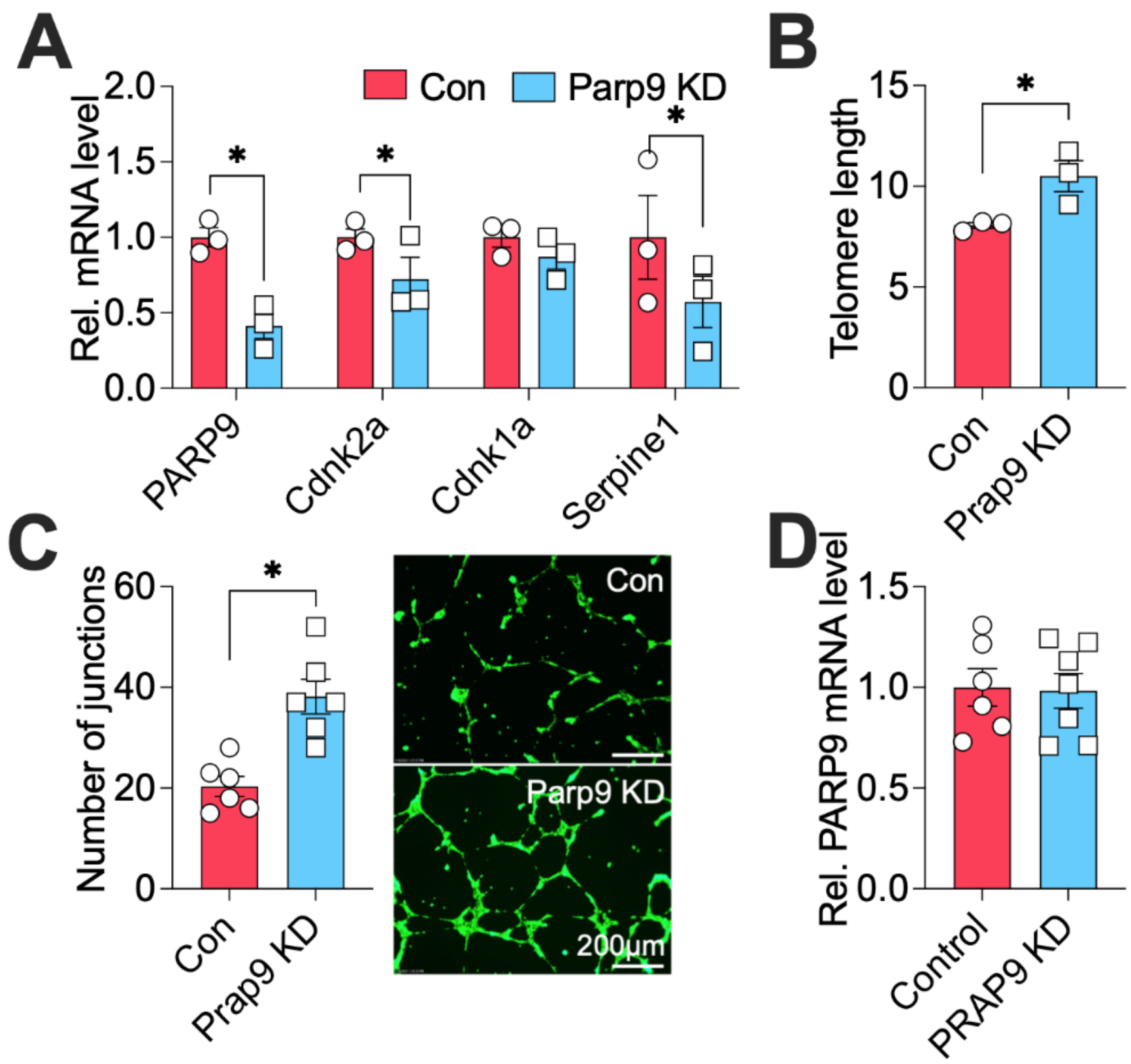

Supplementary Figure S6. PARP9 inhibition attenuates senescence and improves angiogenesis. **A,** Gene expression of PARP9, Cdkn2a, Cdkn1a, and Serpine1 in ECs treated with scrambled control (Con) or PARP9 siRNA (Parp9 KD); n=3 independent biological replicates/group. **B,** Telomere length measured by QRT-PCR; n=3 independent biological replicates/group. **C,** Representative images and quantification of tube formation; n=6 independent biological replicates/group. **D,** PARP9 mRNA expression in the skeletal muscle in mice injected with AAV^{endo}-Control (Control) or AAV^{endo}-PARP9-shRNA (PARP9 KD); n=6-7 independent biological replicates/group. Data shown as mean ± SEM. *p<0.05 by unpaired two-tailed Student's t-test.

SUPPLEMENTARY TABLES

Supplementary Table S1. Gene information of SALTes

SALTe	Gene name	Chr	Start	End	Strand	Ensembl ID	Isoform	Length (bp)	Exon#
1	Gm15564	16	35787121	35803636	-	ENSMUSG00000086324	ENSMUST00000173605	440	4
		16	35787145	35801511	-	ENSMUSG00000086324	ENSMUST00000144134	545	3
		16	35787121	35801770	-	ENSMUSG00000086324	ENSMUST00000174085	831	3
2	1190002F15Rik	6	134905914	134933761	+	ENSMUSG00000098318	ENSMUST00000183867	5803	2
		6	134906061	134928540	+	ENSMUSG00000098318	ENSMUST00000183905	474	3
		6	134905975	134928681	+	ENSMUSG00000098318	ENSMUST00000184504	687	2
		6	134906061	134928306	+	ENSMUSG00000098318	ENSMUST00000184991	243	3
		6	134906093	134928539	+	ENSMUSG00000098318	ENSMUST00000185152	500	3
3	Gm2928	12	109696198	109715703	+	ENSMUSG00000097391	ENSMUST00000181496	1906	11
		12	109696198	109715849	+	ENSMUSG00000097391	ENSMUST00000181543	1300	8
		12	109696199	109697275	+	ENSMUSG00000097391	ENSMUST00000182074	850	2
		12	109696198	109715849	+	ENSMUSG00000097391	ENSMUST00000182185	3822	16
		12	109704136	109706495	+	ENSMUSG00000097391	ENSMUST00000182231	360	3
		12	109701415	109710167	+	ENSMUSG00000097391	ENSMUST00000182268	591	5
		12	109696198	109715849	+	ENSMUSG00000097391	ENSMUST00000182300	1677	9
		12	109696198	109715849	+	ENSMUSG00000097391	ENSMUST00000182499	3188	15
		12	109709926	109715895	+	ENSMUSG00000097391	ENSMUST00000183068	946	4

		12	109696198	109715849	+	ENSMUSG00000097391	ENSMUST00000183084	1553	10
		12	109701419	109714617	+	ENSMUSG00000097391	ENSMUST00000183116	898	7
		12	109696198	109715898	+	ENSMUSG00000097391	ENSMUST00000183144	3797	17
4	Gm45670	7	133349280	133354769	+	ENSMUSG00000109635	ENSMUST00000335131	804	2
5	2310015A16Rik	17	32126853	32128003	-	ENSMUSG00000117879	ENSMUST00000235350	773	2
6	Gm30735	3	53632833	53640487	+	ENSMUSG00000105168	ENSMUST00000241224	688	4
7	Gm42418	17	40157244	40159092	+	ENSMUSG00000119584	ENSMUST00000240377	1849	1
8	Gm28052	17	29768773	29900986	+	ENSMUSG00000099329	ENSMUST00000150388	902	7
9	Gm49015	14	79383699	79388829	-	ENSMUSG00000115167	ENSMUST00000226853	2704	2
10	Gm48492	13	16543529	16547765	-	ENSMUSG00000113322	ENSMUST00000221385	2705	2

Supplementary Table S2. Echocardiography analysis of cardiac function of young mice treated with AAV-control or AAV-SALTe1

	CON (Male, n=6)	OE (Male, n=6)
IVS;d (mm)	1.17±0.06	1.16±0.04
IVS;s (mm)	1.69±0.07	1.61±0.05
LVID;d (mm)	3.94±0.10	3.96±0.26
LVID;s (mm)	2.74±0.15	2.77±0.24
LVPW;d (mm)	1.02±0.03	1.05±0.04
LVPW;s (mm)	1.86±0.04	1.82±0.04
FS (%)	33.62±1.71	32.57±2.18
E/e’	-17.98±1.18	-27.36±1.38*
HR (BPM)	459.7±12.91	429.3±15.63

IVSs: systolic interventricular septum; IVSd diastolic interventricular septum; LVIDd: left ventricular end diastolic internal dimension; LVIDs: left ventricular end systolic dimension; LVPWd: left ventricular end diastolic posterior wall; LVPWs: left ventricular end systolic posterior wall dimension; FS (%): fractional shortening. *$p<0.05$ by Student’s *t* test. Data are shown as mean±SEM.

Supplementary Table S3. Echocardiography analysis of cardiac function of young and aged mice treated with control-GapmeR or SALTe1-specific GapmeR

	Young (Male, n=8)	Aged+Con GapmeR (Male, n=8)	Aged+SALTe1 GapmeR (Male, n=8)
IVS;d (mm)	1.01±0.04	1.25±0.10*	1.12±0.04*
IVS;s (mm)	1.43±0.07	1.73±0.10*	1.64±0.06*
LVID;d (mm)	3.58±0.21	4.11±0.25*	3.76±0.15#
LVID;s (mm)	2.59±0.21	2.96±0.24*	2.60±0.17#
LVPW;d (mm)	0.83±0.05	0.96±0.09*	1.01±0.06*

LVPW;s (mm)	1.40±0.05	1.62±0.10*	1.60±0.08*
FS (%)	31.47±2.08	27.17±1.69	30.91±1.69
E/e’	-14.68±1010	-201.6±1.61*	-15.40±1.06#
HR (BPM)	472.30±7.99	460.30±9.03	457.20±14.87

IVSs: systolic interventricular septum; IVSd diastolic interventricular septum; LVIDd: left ventricular end diastolic internal dimension; LVIDs: left ventricular end systolic dimension; LVPWd: left ventricular end diastolic posterior wall; LVPWs: left ventricular end systolic posterior wall dimension; FS (%): fractional shortening. **p*<0.05 vs Young, #p<0.05 vs Aged+Con GapmeR by One-way ANOVA with Dunnett’s post hoc test. Data are shown as mean±SEM.

Supplementary Table S4. Echocardiography analysis of cardiac function of aged CAS9 knockin mice treated with AAV-control or AAV-SALTe1-gRNA1

	Con (Male, n=6)	gRNA1 (Male, n=6)
IVS;d (mm)	1.08±0.05	1.08±0.04
IVS;s (mm)	1.48±0.05	1.54±0.05
LVID;d (mm)	3.85±0.10	4.04±0.09
LVID;s (mm)	2.86±0.11	2.89±0.09
LVPW;d (mm)	1.07±0.07	1.06±0.08
LVPW;s (mm)	1.60±0.11	1.72±0.07
FS (%)	24.50±0.70	28.43±1.02*
E/e’	-25.56±1.16	-20.11±1.09*
HR (BPM)	488.50±4.72	483.50±16.53

IVSs: systolic interventricular septum; IVSd diastolic interventricular septum; LVIDd: left ventricular end diastolic internal dimension; LVIDs: left ventricular end systolic dimension; LVPWd: left ventricular end diastolic posterior wall; LVPWs: left ventricular end systolic posterior wall dimension; FS (%): fractional shortening. **p*<0.05 by Student’s *t* test. Data are shown as mean±SEM.

Supplementary Table S5. Echocardiography analysis of cardiac function of aged mice treated with AAV-scramble or AAV-PARP9-shRNA

	Control (Male, n=7)	PARP9 shRNA (Male, n=6)
IVS;d (mm)	1.05±0.05	1.16±0.13
IVS;s (mm)	1.49±0.07	1.57±0.07
LVID;d (mm)	3.86±0.16	4.01±0.30
LVID;s (mm)	2.72±0.18	2.67±0.14
LVPW;d (mm)	1.08±0.04	0.98±0.06
LVPW;s (mm)	1.69±0.06	1.67±0.07
FS (%)	29.62±1.33	33.10±1.91
E/e’	-27.11±1.48	-23.35±0.79*
HR (BPM)	442.1±41.24	479.1±19.97

IVSs: systolic interventricular septum; IVSd diastolic interventricular septum; LVIDd: left ventricular end diastolic internal dimension; LVIDs: left ventricular end systolic dimension; LVPWd: left ventricular end diastolic posterior wall; LVPWs: left ventricular end systolic posterior wall dimension; FS (%): fractional shortening. *$p<0.05$ by Student’s *t* test. Data are shown as mean±SEM.

Supplementary Table S6. List of primers used in QPCR and guide RNA sequencing used in CRISPER/dCAS9 system

Primer name	Forward	Reverse
hSALTE1	GGCTTCTAGAACTTGAGCGC	CCAGGAGTCTACGCACAGTT
hPARP9	AGACAGTAGCAATTCCAGCC	GGGTCCTCATTGCTCACCAG
hGAPDH	GGAGCGAGATCCCTCCAAAAT	GGCTGTTGTCATACTTCTCATGG
h/mU6	CTCGCTTCGGCAGCACA	AACGCTTCACGAATTTGCGT
hACTB	CATGTACGTTGCTATCCAGGC	CTCCTTAATGTCACGCACGAT
hRPS18	CAACACCAACATCGATGGGC	GGTGATCACACGTTCCACCT
hHOTAIR	CGGGACTTAGACCCTCAGGT	GTTCCATTCCACTGCGAAGC
hCdkn2a	CCGAATAGTTACGGTCGGAGG	CACCAGCGTGTCCAGGAAG
hCdkn1a	CAGGAGACCTCTAAAGACCCC	GGCTCAGAGAAGTCTGGTGAC
hTP53	AAGTCTAGAGCCACCGTCCA	GACGCTAGGATCTGACTGCG
mSALTe1	CAAGCGTTGGATTGTTCAC	GTATCTGATGCCCTCTTCTG
mRPS18	CATGCAGAACCCACGACAGTA	CCTCACGCAGCTTGTTGTCTA
mGAPDH	ACAGCAACAGGGTGGTGGAC	TTTGAGGGTGCAGCGAACTT
mACTB	GGCTGTATTCCCCTCCATCG	CCAGTTGGTAACAATGCCATGT
mHotair	GCGCCAACGTAGACCAAAAG	TACCGATGTTGGGGACCTCT
mPARP9	AGGACGCCAAAGGGATCTG	CCGGCTCCATAAACTGGGT
mKPNA1	ACCAGGAAAAGAGAACTTTCGC	GTCAGAGGTGATGACACCCC
mSALTe2	TTCCTAAGCCATCAGGAG	GAGACAGAAGGACACAGGG
mSALTe3	ACCCACCATCATCAGTGTC	GGTCACACCATTAGCAAGC
mSALTe4	GGCCTCTGATAGACAGCTAACAC	GCAGCAATGCATAACGGGCTTC
mSALTe5	CCTTCCAGCTCCCTTTTACCATC	GGATGCATAGAAACAAGGAAGG
mSALTe6	GGAGGACTGATAAGCAGCACGG	CTTCAGCATAAGAGCTGGATCC
mSALTe7	GGTTTCCGTAGGTGAACCTGCG	CGGACCCAGACCGACCCCACC
mSALTe8	GCAGCAAAAGCATCTGCTACAAG	GGAGGTCTCCACGATGTCCTTCC
mSALTe9	CGCCTGCTTGCTGCGTGAGACTG	GTGTTAATTTGCTCAAGTAAGG
mSALTe10	GGCACAAGCAAGTTACATGAAG	GTAATGCTGCAGCTGTCCACTTC

mChaer	TCCAATGAGGGAAGCGAAGC	GTCCGATGCCAGTTCCAGTT
mIL-6	TAGTCCTTCCTACCCCAATTTCC	TTGGTCCTTAGCCACTCCTTC
mIL-1β	GCAACTGTTCCTGAACTCAACT	ATCTTTTGGGGTCCGTCAACT
mTNFα	CCCTCACACTCAGATCATCTTCT	GCTACGACGTGGGCTACAG
mCCL2	TTAAAAACCTGGATCGGAACCAA	GCATTAGCTTCAGATTTACGGGT
mVCAM1	AGTTGGGGATTCGGTTGTTCT	CCCCTCATTCCTTACCACCC
mICAM1	GTGATGCTCAGGTATCCATCCA	CACAGTTCTCAAAGCACAGCG
mANGPT1	CACATAGGGTGCAGCAACCA	CGTCGTGTTCTGGAAGAATGA
mPDGFA	GAGGAAGCCGAGATACCCC	TGCTGTGGATCTGACTTCGAG
mVEGFa	GCACATAGAGAGAATGAGCTTCC	CTCCGCTCTGAACAAGGCT
mHIF1a	ACCTTCATCGGAAACTCCAAAG	ACTGTTAGGCTCAGGTGAACT
mTIE-2	GAGTCAGCTTGCTCCTTTATGG	AGACACAAGAGGTAGGGAATTGA
mCdkn2a	GCTCAACTACGGTGCAGATTC	GCACGATGTCTTGATGTCCC
mCdkn1a	CGAGAACGGTGGAACTTTGAC	CCAGGGCTCAGGTAGACCTT
mTP53	ATCGCCTTCGACATCATCGC	CCCCATGCGTACTCCATGAG
mSeripine1	CACAGGCACTGCAAAAGGTC	TGTGCCGAACCACAAAGAGA
mNPPA	GTAGGATTGACAGGATTGGA	TCCTCCTTGGCTGTTATC
mNPPB	GCTGCTGGAGCTGATAAGAGAA	GTTCTTTTGTAGGGCCTTGGTC
mNRG1	AGTGCCCAAATGAGTTTACTGG	AGTTCCTCCGCTTCCATAAATTC
mFGF2	TGGTGACCACAAGCTGAATG	TCCCTTGATAGACACAACTCCTC
mIGF-1	GTGAGCCAAAGACACACCCA	ACCTCTGATTTTCCGAGTTGC
Primers for RPA assay		
Non-overlap	CTTAGACAAGCAGGGGTTGAAATG	CAGGACTTCATTGCATCTTTC
Overlap	CAGAGCTGCCATTTAAGACCC	GGCCATGCCCCTGTACTTGTGG
gRNA for CRISPR/dCAS9 system		
Control	CACCGGCACTCACATCGCTACATCA	
SALTe1	CACCGGTCCCGGTGCCACTACGAAG	CACCGGGAGTTGGCAGGTTACCACC
SALTe2	CACCGCAAGTGAGGAGCATCGTCTT	CACCGTGGGACACCCCAATTGCTTT
SALTe9	CACCGCAACCACCAGCTCGGGACGA	CACCGACAACCACCAGCTCGGGACG

Pulldown probe	Antisense probes	Sense probes
	TTCTATATGTCTGCGCTTTT	TCTTATTGTCTTCTCTGCAT
	TCATCCAATAAGCATGCTCG	ACTTAAATTGTTTCCCGCAC
	ATCTACTTGGGAGTAAGGGT	CAGCAGACCTAGAAAGGGAC
	CATAGTGGTTTAACACCCAT	ATTCTTGCTGTCACCTATAG
	ACCTGTTACTTAATTCCTGT	AGACTCAGAACTCCGTAAGC
	AAGCTGTACTCTCCTTGTAA	GGAAAGAGGTTTTATGAATGGTG
	AAGTGTCTCATGTGGCTTAT	CTTTTTTGCCTACATAGCAG
	CTTACTAAGCCATTAGCTGT	CCAATGGGTGTTAAACCACT
	GTTAGAGCCATGCAGAGAAG	CGGCAATTTCCAGCGAAAAT
	TGCCTCAGCCTCCCTGGGGT	GGTTGGCAGTCAGTAAAAGC

Reference

1. Roh JD, Castro C, Yu A, Rana S, Shahul S, Gray KJ, et al. Placental senescence pathophysiology is shared between peripartum cardiomyopathy and preeclampsia in mouse and human. *Sci Transl Med* 2024;**16**:eadi0077. doi: https://doi.org/10.1126/scitranslmed.adi0077
2. Liu X, Xiao J, Zhu H, Wei X, Platt C, Damilano F, et al. miR-222 is necessary for exercise-induced cardiac growth and protects against pathological cardiac remodeling. *Cell Metab* 2015;**21**:584–595. doi: https://doi.org/10.1016/j.cmet.2015.02.014
3. Li H, Trager LE, Liu X, Hastings MH, Xiao C, Guerra J, et al. lncExACT1 and DCHS2 Regulate Physiological and Pathological Cardiac Growth. *Circulation* 2022;**145**:1218–1233. doi: https://doi.org/10.1161/CIRCULATIONAHA.121.056850
4. Vujic A, Lerchenmuller C, Wu TD, Guillermier C, Rabolli CP, Gonzalez E, et al. Exercise induces new cardiomyocyte generation in the adult mammalian heart. *Nat Commun* 2018;**9**:1659. doi: https://doi.org/10.1038/s41467-018-04083-1
5. Wang C, Shen S, Kang J, Sugai-Munson A, Xiao X, Zhang Y, et al. METTL3 Is Essential for Exercise Benefits in Diabetic Cardiomyopathy. *Circulation* 2025. doi: https://doi.org/10.1161/CIRCULATIONAHA.124.070279
6. Roh JD, Houstis N, Yu A, Chang B, Yeri A, Li H, et al. Exercise training reverses cardiac aging phenotypes associated with heart failure with preserved ejection fraction in male mice. *Aging Cell* 2020;**19**:e13159. doi: https://doi.org/10.1111/acel.13159
7. Bezzerides VJ, Platt C, Lerchenmuller C, Paruchuri K, Oh NL, Xiao C, et al. CITED4 induces physiologic hypertrophy and promotes functional recovery after ischemic injury. *JCI Insight* 2016;**1**. doi: https://doi.org/10.1172/jci.insight.85904
8. Roh JD, Hobson R, Chaudhari V, Quintero P, Yeri A, Benson M, et al. Activin type II receptor signaling in cardiac aging and heart failure. *Sci Transl Med* 2019;**11**. doi: https://doi.org/10.1126/scitranslmed.aau8680

9. Yucel N, Axsom J, Yang Y, Li L, Rhoades JH, Arany Z. Cardiac endothelial cells maintain open chromatin and expression of cardiomyocyte myofibrillar genes. *Elife* 2020;**9**. doi: https://doi.org/10.7554/eLife.55730
10. Sanjana NE, Shalem O, Zhang F. Improved vectors and genome-wide libraries for CRISPR screening. *Nat Methods* 2014;**11**:783–784. doi: https://doi.org/10.1038/nmeth.3047
11. Konermann S, Brigham MD, Trevino AE, Joung J, Abudayyeh OO, Barcena C, et al. Genome-scale transcriptional activation by an engineered CRISPR-Cas9 complex. *Nature* 2015;**517**:583–588. doi: https://doi.org/10.1038/nature14136
12. Torres M, Becquet D, Guillen S, Boyer B, Moreno M, Blanchard MP, et al. RNA Pull-down Procedure to Identify RNA Targets of a Long Non-coding RNA. *J Vis Exp* 2018. doi: https://doi.org/10.3791/57379
13. Jiang J, Wakimoto H, Seidman JG, Seidman CE. Allele-specific silencing of mutant Myh6 transcripts in mice suppresses hypertrophic cardiomyopathy. *Science* 2013;**342**:111–114. doi: https://doi.org/10.1126/science.1236921
14. Liu YB, Xu BC, Chen YT, Yuan X, Liu JY, Liu T, et al. Directed evolution of AAV accounting for long-term and enhanced transduction of cardiovascular endothelial cells in vivo. *Mol Ther Methods Clin Dev* 2021;**22**:148–161. doi: https://doi.org/10.1016/j.omtm.2021.05.015
15. Pleger ST, Shan C, Ksienzyk J, Bekeredjian R, Boekstegers P, Hinkel R, et al. Cardiac AAV9-S100A1 gene therapy rescues post-ischemic heart failure in a preclinical large animal model. *Sci Transl Med* 2011;**3**:92ra64. doi: https://doi.org/10.1126/scitranslmed.3002097
16. Guo Y, VanDusen NJ, Zhang L, Gu W, Sethi I, Guatimosim S, et al. Analysis of Cardiac Myocyte Maturation Using CASAAV, a Platform for Rapid Dissection of Cardiac Myocyte Gene Function In Vivo. *Circ Res* 2017;**120**:1874–1888. doi: https://doi.org/10.1161/CIRCRESAHA.116.310283
